# Algorithms for the analysis of ensemble neural spiking activity using simultaneous-event multivariate point-process models

**DOI:** 10.3389/fncom.2014.00006

**Published:** 2014-02-10

**Authors:** Demba Ba, Simona Temereanca, Emery N. Brown

**Affiliations:** ^1^Department of Anesthesia, Critical Care and Pain Medicine, Harvard Medical School, Massachusetts General HospitalCharlestown, MA, USA; ^2^Department of Brain and Cognitive Sciences, Massachusetts Institute of TechnologyCambridge, MA, USA; ^3^Athinoula A. Martinos Center for Biomedical Imaging, Harvard Medical School, Massachusetts General HospitalCharlestown, MA, USA; ^4^Institute for Medical Engineering and Science, Massachusetts Institute of TechnologyCambridge, MA, USA

**Keywords:** multivariate point-process, simultaneous events, multinomial GLM, thalamic synchrony

## Abstract

Understanding how ensembles of neurons represent and transmit information in the patterns of their joint spiking activity is a fundamental question in computational neuroscience. At present, analyses of spiking activity from neuronal ensembles are limited because multivariate point process (MPP) models cannot represent simultaneous occurrences of spike events at an arbitrarily small time resolution. Solo recently reported a simultaneous-event multivariate point process (SEMPP) model to correct this key limitation. In this paper, we show how Solo's discrete-time formulation of the SEMPP model can be efficiently fit to ensemble neural spiking activity using a multinomial generalized linear model (mGLM). Unlike existing approximate procedures for fitting the discrete-time SEMPP model, the mGLM is an exact algorithm. The MPP time-rescaling theorem can be used to assess model goodness-of-fit. We also derive a new marked point-process (MkPP) representation of the SEMPP model that leads to new thinning and time-rescaling algorithms for simulating an SEMPP stochastic process. These algorithms are much simpler than multivariate extensions of algorithms for simulating a univariate point process, and could not be arrived at without the MkPP representation. We illustrate the versatility of the SEMPP model by analyzing neural spiking activity from pairs of simultaneously-recorded rat thalamic neurons stimulated by periodic whisker deflections, and by simulating SEMPP data. In the data analysis example, the SEMPP model demonstrates that whisker motion significantly modulates simultaneous spiking activity at the 1 ms time scale and that the stimulus effect is more than one order of magnitude greater for simultaneous activity compared with non-simultaneous activity. Together, the mGLM, the MPP time-rescaling theorem and the MkPP representation of the SEMPP model offer a theoretically sound, practical tool for measuring joint spiking propensity in a neuronal ensemble.

## Introduction

The study of how neurons represent and transmit information in the patterns of their ensemble spiking activity has been greatly facilitated in the last 17 years by the technical capability to measure simultaneously multiple single neuron activity using multiple electrode recording techniques (Wilson and McNaughton, 1993; Maynard et al., 1997). For this reason, the development of statistical methods to characterize ensemble spiking activity is an active field of neuroscience research (Brown et al., 2004; Grün, 2009).

Several histogram-based methods have been used to analyze ensemble neural spiking activity. These include the cross-correlogram (Brody, 1999), the cross-intensity function (Brillinger et al., 1976) and the joint peristimulus time histogram (Gerstein and Perkel, 1969). An appeal of these methods is that, as discrete approximations to well-known statistics used to analyze continuous-valued processes, they are easy to understand and to compute. However, like all histogram methods, large sample theory is required to justify this approach (Klemelä, 2009). Furthermore, these methods only measure the association between pairs of neurons and the fundamental assumption of stationarity that underlies their construction can be hard to justify given that neural spiking activity is often highly plastic and adaptive.

Algorithms to detect precise patterns of spike timing are another method of measuring associations among neural spike trains (Grün et al., 2002; Gütig et al., 2002; Pipa and Grün, 2002; Grün, 2009). The appeal of these methods is that they offer a way to evaluate higher-order neural interactions in ensemble spiking activity beyond pairwise comparisons. These methods require the user to specify the complexity of the pattern to be analyzed and assume that, at least approximately, neural systems use repeatedly the same patterns to represent and transmit information. Frequency domain estimates of coherence which also require a stationarity assumption (Brillinger, 1992; Mitra and Bokil, 2008) and gravity clustering, a high-dimensional graphical technique, Lindsey and Gerstein (2006) have also been used to analyze joint spiking activity. A common paradigm in neurophysiology is to apply a stimulus and to observe the response of the neural ensemble. The methods cited thus far have limited ability to relate the effects of the stimulus to the ensemble response.

Likelihood methods using either an information-geometric (Nakahara and Amari, 2002; Amari and Nakahara, 2006; Shimazaki et al., 2012) or a point-process (Ogata, 1981; Chornoboy et al., 1988; Okatan et al., 2005) representation provide an alternative parametric model-based approach to analyzing ensemble neural spiking activity. Likelihood methods can relate the ensemble activity to any relevant covariates. When the parametric model accurately describes the data, these analyses have important optimality properties. However, this approach has an important shortcoming that is especially relevant for analysis of coincident spiking activity. At an arbitrarily small time scale these methods do not allow simultaneous spiking (Ogata, 1981; Karr, 1991; Daley and Vere-Jones, 2003). Current analysis of spiking activity from neuronal ensembles circumvent this limitation by either assuming neurons are independent conditioned on history, or simply ignoring simultaneous events. Ventura et al. (2005) developed a likelihood procedure to overcome this limitation for analyzing a pair of neurons. In Kass et al. (2011), Kass et al. extend Ventura's approach to multiple neurons. Solo recently reported a simultaneous-event multivariate point process (SEMPP) model to correct this key limitation in general (Solo, 2007).

In this paper, we propose a multinomial generalized linear model (mGLM) of a discrete-time formulation of the SEMPP, which can be efficiently fit to ensemble neural spiking activity, and derive a new marked point-process (MkPP) representation of the SEMPP model. We focus on the important, non-trivial, algorithmic implications and properties of the mGLM and the MkPP representation. Existing procedures for fitting a GLM to discrete-time SEMPP data (Chornoboy et al., 1988; Okatan et al., 2005) are approximate ones which, conditioned on history, fit separate GLMs to each component of the SEMPP. In contrast, the mGLM algorithm is an exact procedure which fits a joint model to the discrete-time SEMPP data. We use the multivariate point process (MPP) time-rescaling theorem (Daley and Vere-Jones, 2003; Vere-Jones and Schoenberg, 2004) to assess model goodness-of-fit. The MkPP representation and its implications are not trivial consequences of the treatment in Solo (2007). In particular, it leads to new thinning and time-rescaling algorithms for efficiently simulating an SEMPP stochastic process. These algorithms are much simpler than those based on obvious extensions to SEMPPs of algorithms for simulating a univariate point-process (Brown et al., 2002). We illustrate the new SEMPP model representation by analyzing neural spiking activity from pairs of simultaneously-recorded rat thalamic neurons stimulated by periodic whisker deflections and by simulating SEMPP data.

## Methods

In this section, we develop the theory behind our algorithms for handling simultaneous events in MPP. We propose a statistical model, based on generalized linear models, to analyze simultaneous recordings from pairs of thalamic neurons. Unlike existing procedures, the algorithm for fitting this mGLM estimates the parameters of interest jointly, using the discrete-time multivariate SEMPP data. We also introduce a new MkPP representation of SEMPP data and highlight its implications for simulation of such data.

### Theory

#### Simultaneous-event multivariate point processes

In Solo (2007), Solo lays out a theoretical SEMPP model. In order to develop our algorithms, we review the essential features of Solo's model. We also show how one can derive the joint probability density function (PDF) of an SEMPP in discrete and continuous time using straightforward heuristic arguments.

We consider an observation interval (0, *T*] and, for *t* ∈ (0, *T*], let *N*(*t*) = (*N*_1_(*t*), *N*_2_(*t*), …, *N*_*C*_(*t*))′ be a *C*-variate point-process defined as *N*_*c*_(*t*) = $N_{c}(t)=\int_{0}^{t}dN_{c}(u)$, where *dN*_*c*_(*t*) is the indicator function which is 1 if there is an event at time *t* and 0 otherwise, for *c* = 1, …, *C*. *N*_*c*_(*t*) counts the number of events for component *c* in the interval (0, *t*]. We assume that each component *c* has a conditional intensity function (CIF) defined as

(1) $$\lambda_{c}(t|H_{t})=\underset{\Delta \to 0}{\lim}\frac{P[N_{c}(t+\Delta )-N_{c}(t)=1|H_{t}]}{\Delta },$$

where *H*_*t*_ is the history of the *C*-variate point process up to time *t*. Let *dN*(*t*) = (*dN*_1_(*t*), *dN*_2_(*t*), …, *dN*_*C*_(*t*))′ be the vector of indicator functions *dN*_*c*_(*t*) at time *t*. We may treat *dN*(*t*) as a *C*-bit binary number. Therefore, there are 2^*C*^ possible outcomes of *dN*(*t*) at any *t*. *C* of these outcomes have only one non-zero bit [that is, only one event in one component of *dN*(*t*)] and 2^*C*^−*C*−1 have two or more non-zero bits. That is, there is an event at time *t* in at least two components of *dN*(*t*). The last outcome is *dN*(*t*) = (0, …, 0)′.

We define *N*(*t*) as a SEMPP if, at any time *t*, *dN*(*t*) has at least two non-zero bits. That is, events are observed simultaneously in at least two of the components of *N*(*t*). The special case in which, at any *t*, *dN*(*t*) can only take as values one of the *C* outcomes for which only one of the bits of *dN*(*t*) is non-zero is the MPP defined by Daley and Vere-Jones (2003). The joint probability density of *N*(*t*) in this special case is given by the Jacod likelihood function (Ogata, 1981; Karr, 1991; Daley and Vere-Jones, 2003).

#### The disjoint and marked point process representations

To derive the joint probability density function of an SEMPP, we develop, in a fashion similar to Solo (2007), an alternative representation of *N*(*t*). Let *M* = 2^*C*^ be the number of possible outcomes of *dN*(*t*) at *t*. We define a new *M* − 1-variate point process N^*^(*t*) = (N^*^_1_(*t*), N^*^_2_(*t*), …, N^*^_M − 1_(*t*))′ of disjoint outcomes of *N*(*t*). That is, each component of N^*^(*t*) is a counting process for one and only one of the 2^*C*^−1 outcomes of *dN*(*t*) (patterns of *C* bits) that have at least one non-zero bit. For any *t*, the vector dN^*^(*t*) = (dN^*^_1_(*t*), …, dN^*^_M − 1_(*t*))′ is a *M*−1-bit binary number with at most one non-zero bit. The non-zero element of dN^*^(*t*) (if any) is an indicator of the pattern *dN*(*t*) of *C* bits which occurs at *t*. dN^*^(*t*) = (0, …, 0)′ corresponds to *dN*(*t*) = (0, …, 0)′. We define the CIF of N^*^_m_(*t*) as

(2) $$\lambda_{m}^{*}(t|H_{t})=\underset{\Delta \to 0}{\lim}\frac{P\left[N_{m}^{*}(t+\Delta )-N_{m}^{*}(t)=1|H_{t}\right]}{\Delta },$$

where the counting process is N^*^_m_(*t*) = $N_{m}^{*}(t)=\int_{0}^{t}dN_{m}^{*}(u)$. We term N^*^(*t*) the *disjoint* process or representation.

One simple way to map from *dN*(*t*) to dN^*^(*t*) is to treat the former as a *C*-bit binary number, reverse the order of its bits, and convert the resulting binary number to a decimal number. We use this decimal number as the index of the non-zero component of dN^*^(*t*). The inverse map proceeds by finding the index of the non-zero entry of dN^*^(*t*), expressing this index as a *C*-bit binary number, and reversing the order of the bits to obtain *dN*(*t*). This one-to-one map is described in detail in Supplemental Material 1 for the arbitrary *C*-variate case. Table 1 illustrates this one-to-one map for the case *C* = 3 and *M* = 8. In this example, *N*(*t*) is related to N^*^(*t*) by

(3) $$N_{1}(t)=N_{1}^{*}(t)+N_{3}^{*}(t)+N_{5}^{*}(t)+N_{7}^{*}(t)$$

(4) $$N_{2}(t)=N_{2}^{*}(t)+N_{3}^{*}(t)+N_{6}^{*}(t)+N_{7}^{*}(t)$$

(5) $$N_{3}(t)=N_{4}^{*}(t)+N_{5}^{*}(t)+N_{6}^{*}(t)+N_{7}^{*}(t).$$

**Table 1:** Map from *dN(t)* to dN*(*t*), *C* = 3, *M* = 8.

***dN*(*t*)**	***m***	dN*(*t*)
(1,0,0)	1	(1,0,0,0,0,0,0)
(0,1,0)	2	(0,1,0,0,0,0,0)
(1,1,0)	3	(0,0,1,0,0,0,0)
(0,0,1)	4	(0,0,0,1,0,0,0)
(1,0,1)	5	(0,0,0,0,1,0,0)
(0,1,1)	6	(0,0,0,0,0,1,0)
(1,1,1)	7	(0,0,0,0,0,0,1)

The CIFs of *N*(*t*) are related to those of N^*^(*t*) in a similar manner.

If we let 0 < *t*_1_ < *t*_2_ < … < *t*_*L*_ ≤ *T* denote the times in the observation interval (0, *T*] at which *dN*(*t*) has at least one non-zero bit, then we can express the disjoint process N^*^(*t*) as a MkPP {(*t*_ℓ_, dN^*^(*t*_ℓ_)}^*L*^_ℓ = 1_. At *t*_ℓ_, at least one of the bits of *dN*(*t*) is non-zero. The non-zero bit of dN^*^(*t*_ℓ_) then indicates, through the map described in Supplemental Material 1, exactly which of the *M*−1 patterns of *C* bits (outcomes of *dN*(*t*) other than (0, …, 0)′) occurred at *t*_ℓ_. At any other *t*, *dN*(*t*) = (0, …, 0)′. We term the unmarked process {*t*_ℓ_}^*L*^_ℓ = 1_ the ground point process (Daley and Vere-Jones, 2003) and denote by *dN*_*g*_(*t*) the indicator function that is 1 at *t*_ℓ_, ℓ = 1, …, *L* and zero at any other *t*. The ground point process defines the times of occurrence of *any* pattern of *C* bits (outcomes of *dN*(*t*)) that are not all zero. For each *m*, the times at which dN^*^_m_(*t*) is non-zero define the times of occurrence of *one specific* pattern of *C* bits that are not all zero. It follows that the counting process and the CIF of the ground point process are respectively

(6) $$N_{g}(t)=\sum_{m=1}^{M-1}N_{m}^{*}(t)$$

(7) $$\lambda_{g}^{*}(t|H_{t})=\sum_{m=1}^{M-1}\lambda_{m}^{*}(t|H_{t}).$$

The probability of the marks is given by the multinomial probability mass function

(8) $$P[dN_{m}^{*}(t)=1|dN_{g}(t)=1,H_{t}]=\frac{\lambda_{m}^{*}(t|H_{t})}{\lambda_{g}^{*}(t|H_{t})},$$

for *m* = 1, …, *M*−1. The derivation of Equations (7, 8) are in Supplemental Material 1, on the Frontiers website. The MkPP representation provides an efficient description of *N*(*t*). The probability of an event occurring in (0, *T*] is governed by the CIF λ^*^_g_(*t*|*H*_*t*_) of the ground point process. When an event is observed in *dN*_*g*_(*t*), the marks are drawn from an *M*−1-dimensional history-dependent multinomial distribution (Equation 8) to produce the corresponding event in N^*^(*t*), or equivalently *N*(*t*).

#### Three algorithmically-useful forms of the joint probability density function

We give three forms of the joint PDF of an SEMPP. The derivations for these PDFs follow from simple heuristic arguments, which we detail in Supplemental Material 1. Here, we focus on the algorithmic importance of these forms.

First, we give the joint PDF on an SEMPP in discrete-time, which is the basis for the mGLM algorithm used in **Data Analysis**. Then, we give the continuous PDF of the disjoint representation, from which a multivariate analog of the time-rescaling theorem and a Kolmogorov–Smirnov (KS) test for goodness-of-fit assessment can be deduced (Brown et al., 2002; Daley and Vere-Jones, 2003). Lastly, we express the continuous PDF in terms of the MkPP representation. This latter form leads to simple algorithms for simulating an SEMPP stochastic process.

We define the discrete-time representations of *N*(*t*) and N^*^(*t*) in Supplemental Material 1. In this notation, ΔN^*^ is the *I* × *M*−1 matrix of discretized outcomes of dN^*^(*t*) for the observation interval (0, *T*]. Each column ΔN^*^_i_ of ΔN^*^, where *i* is the discrete-time index, is a realization from a multinomial trial with *M* outcomes (roll of an *M*-sided die). The probability mass function of ΔN^*^ can be written as the product of conditional *M*-nomial trials:

(9) $$\begin{array}{l} P[\Delta N^{*}]=\prod_{i=1}^{I}\prod_{m=1}^{M-1}{\left(\lambda_{m}^{*}[i|H_{i}]\Delta \right)}^{\Delta N_{m,i}^{*}}\\ \;\cdot {\left(1-\lambda_{g}^{*}[i|H_{i}]\Delta \right)}^{1-\Delta N_{g,i}}+o(\Delta^{L}). \end{array}$$

We can obtain the continuous-time joint PDF *p*[N^*^_(0, *T*]_] of the disjoint process N^*^(*t*) by relating it to the discrete-time joint PDF and then taking limits. This leads to *p*[N^*^_(0, *T*]_] being expressed as the product of *M*−1 continuous-time univariate point process PDFs

(10) $$\begin{array}{l} p[N_{\left(0,T\right]}^{*}]=\prod_{m=1}^{M-1}\exp\left\{\int_{0}^{T}\log\lambda_{m}^{*}(t|H_{t})dN_{m}^{*}(t)\right\}\\ \;\cdot \text{exp}\left\{-\int_{0}^{T}\lambda_{m}^{*}(t|H_{t})dt\right\}. \end{array}$$

The PDF *p*[N^*^_(0, *T*]_] can also be written in terms of the MkPP representation as

(11) $$\begin{array}{l} p[N_{\left(0,T\right]}^{*}]=\prod_{\ell =1}^{L}\prod_{m=1}^{M-1}{\left(\frac{\lambda_{m}^{*}(t_{\ell }|H_{t_{\ell }})}{\lambda_{g}^{*}(t_{\ell }|H_{t_{\ell }})}\right)}^{dN_{m}^{*}(t_{\ell })}\\ \;\cdot \lambda_{g}^{*}{\left(t_{\ell }|H_{t_{\ell }}\right)}^{dN_{g}(t_{\ell })}\text{exp}\left\{-\int_{0}^{T}\lambda_{g}^{*}(t|H_{t})dt\right\}\text{​}. \end{array}$$

We use the discrete-time representation (Equation 9) to form the mGLM (Fahrmeir and Tutz, 2001) to analyze the joint spiking activity of a pair of thalamic neurons in **Data Analysis**. Existing procedures for fitting a GLM to discrete-time SEMPP data (Chornoboy et al., 1988; Okatan et al., 2005) are approximate ones which, conditioned on history, fit separate GLMs to each component of the disjoint representation of the SEMPP. In contrast, the mGLM algorithm is an exact procedure which jointly estimates the parameters of the model using the multivariate discrete-time SEMPP data.

The continuous-time PDF of the disjoint representation (Equation 10) is the form derived by Solo (2007). If we let N^*^(*t*) be the MPP defined by restricting dN^*^(*t*) to the *C* components which are indicators for the outcomes for which only one bit of *dN*(*t*) is non-zero (that is, if we disregard simultaneous occurrence of events), then Equation (10) gives the joint PDF of the MPP defined by the Jacod likelihood which has no simultaneous events (Chornoboy et al., 1988; Karr, 1991; Okatan et al., 2005). The case *M* = 2 corresponds to the joint PDF of a univariate point process (Truccolo et al., 2005). N^*^(*t*) is an *M*−1-variate MPP because it is composed of the disjoint events of *dN*(*t*). Therefore, by Proposition 7.4.VI in Daley and Vere-Jones (2003), it can be transformed into an *M*−1-variate point process with independent unit-rate Poisson processes as its components. This observation, applied to Equation (10), allows us to create a multivariate extension of the KS plots to assess goodness-of-fit of SEMPP models fit to simultaneous neural spiking activity.

Finally, Equation (11) makes explicit the formulation of the joint PDF of an SEMPP as an MkPP. The PDF of Equation (11) follows from the observation that, in any small time interval Δ, the SEMPP is a multinomial model with 2^*C*^ possible outcomes (Equation 9, *M* = 2^*C*^). A multinomial model with 2^*C*^ outcomes can be written as the product of a binomial probability model and a conditional multinomial probability model with 2^*C*^−1 outcomes. The binomial probability model defines the ground process and the conditional multinomial process defines the marked process. The CIF of the ground point process is given in Equation (7) and the mark process is defined by the history-dependent *M*−1-dimensional multinomial distribution in Equation (8). The intuition behind the MkPP representation, as well as its implications, should not be dismissed as a trivial consequences of the treatment in Solo (2007). An important consequence of the MkPP representation is that it can be combined with either a thinning algorithm or the univariate time-rescaling theorem (Brown et al., 2002) to yield highly efficient simulation algorithms for SEMPP models. As we shall see, these algorithms are much simpler than those based on obvious extensions to SEMPPs of algorithms for simulating a univariate point-process (Brown et al., 2002). Indeed, the simulation algorithms which use the MkPP representation make simulation of SEMPP data almost as simple as simulation of a univariate point process.

Details of the derivations for Equations (9–11) are in Supplemental Material 1. The derivation for Equation (10) follows from simple heuristic arguments used previously in Truccolo et al. (2005) for univariate point processes and in Solo (2007) for SEMPPs. The key idea is to start with a discrete-time form, as in Equation (9), and then take limits.

#### Simulating simultaneous-event multivariate point processes

For univariate point processes, an important consequence of the time-rescaling theorem is that it leads to an algorithm for simulating univariate point-process data (Brown et al., 2002). The time-rescaling theorem for MPPs also leads to an algorithm for simulating SEMPP data, which we describe below. In the absence of history dependence of the CIFs, this algorithm is a trivial extension of the one for simulating a point process using the univariate time-rescaling theorem (Brown et al., 2002). For reasons detailed below, this algorithm, which uses the disjoint representation, becomes unwieldy in the more interesting case where the CIFs depend on the history of the SEMPP. In contrast, using the MkPP representation leads to two algorithms (also described below), which make the task of simulating SEMPP data almost as simple as simulating a univariate point process.

**Algorithm 1 (Multivariate time-rescaling):**

1. Set *t*_0_ = 0, ℓ = 1, ℓ_*m*_ = 1 ∀ *m* ∈ {1, …, *M* − 1}.
2. ∀ *m*, draw τ_ℓ_*m*__ an exponential random variable with mean 1.
3. ∀ *m*, find *t*_ℓ_*m*__ as the solution to:
  $\tau_{\ell_{m}}=\int_{t_{\ell_{m}-1}}^{t_{\ell_{m}}}\lambda_{m}^{*}(t|H_{t})dt$.
  Let *m*^+^ = arg min_*m*_ *t*_ℓ_*m*__, *t*_ℓ_ = *t*_ℓ_*m*^+^__.
4. If *t*_ℓ_ > *T*, then stop the algorithm, else
5. If *m* = *m*^+^, set dN^*^_m^+^_(*t*_ℓ_) = 1, ℓ_*m*_ = ℓ_*m*_ + 1 and draw τ_ℓ_*m*__ an exponential random variable with mean 1.
6. If *m* ≠ *m*^+^, ℓ_*m*_ does not change, set
  $\tau_{\ell_{m}}=\tau_{\ell_{m}}-\int_{t_{\ell_{m}-1}}^{t_{\ell }}\lambda_{m}^{*}(t|H_{t})dt$,
  *t*_ℓ_*m* − 1__ = *t*_ℓ_,
  dN^*^_m_(*t*_ℓ_) = 0,
7. *dN*(*t*_ℓ_) is obtained from dN^*^(*t*_ℓ_) using the map described in Supplemental Material 1.
8. ℓ = ℓ + 1.
9. Go back to 3.

Although Algorithm 1 is not easy to parse, we describe the intuition behind it, which follows from the time-rescaling theorem for MPPs. Step 2 of the algorithm prescribes that we should first generate independent exponential random variables with mean 1, one for each component of the disjoint representation of the SEMPP. Then, for each component, we find in Step 3 the times at which the area under each CIF integrates to the value prescribed by the exponential draws. If the CIFs did not depend on history, it would be sufficient to repeat these two steps, until we've simulated the SEMPP data up to a given time *T*. In other words, without dependence of the CIFs on history, one can simulate an SEMPP by *separate* simulation of multiple univariate point processes. Intuitively, this is because the integration of the CIFs in Step 3 of Algorithm 1 become decoupled when there is no history dependence, that is, no component of the SEMPP needs to update its own history based on events that occur in the other components. However, in the more interesting case when the CIFs depend on history, Step 3 of Algorithm 1 becomes problematic: integration of the CIFs must be carried out *in parallel* because whenever an event occurs in one component, all processes must update their history accordingly. Moreover, only the process for which an event occurs must perform a new exponential 1 draw (Step 5). All other processes keep computing the area under their CIF until it reaches the value prescribed by their exponential 1 draw (Step 6).

Simply put, when the CIFs depend on history, Step 3 of Algorithm 1 leads to an undesirable amount of bookkeeping, which grows with the number of component of the SEMPP.

Algorithm 1 should be contrasted with one which uses the MkPP representation. In this algorithm, we simulate events from the ground process (which is a univariate point process) using the univariate time-rescaling theorem, e.g., as in Brown et al. (2002). When an event occurs in the ground process, we roll an *M*−1-sided die to decide to which component of the disjoint process this event should be assigned. The key feature of this algorithm, which makes it simpler and more elegant than Algorithm 1, is that we must perform only one uniform draw at each step. The components of the SEMPP only interact with each other, through the mark pmf, when an event occurs in the ground process. This makes all of the bookkeeping of Algorithm 1 unnecessary.

The following algorithm for simulating an SEMPP based on the MkPP representation is an extension of the thinning simulation algorithm for MPP models developed by Ogata (1981).

**Algorithm 2 (Thinning):** Suppose there exists λ such that λ^*^_g_(*t*|*H*_*t*_) ≤ λ for all *t* ∈ (0, *T*]:

1. Simulate observations 0 < *t*_1_ < *t*_2_ … < *t*_*K*_ ≤ *T* from a Poisson point process with rate λ.
2. Set *k* = 1.
3. while *k* ≤ *K*
  - Draw *u*_*k*_ from the uniform distribution on (0,1)
  - if $\frac{\lambda_{g}^{*}(t_{k}|H_{t_{k}})}{\lambda }\geq u_{k}$
    - Draw *m*_*k*_ from the (*M*−1)-dimensional multinomial distribution with probabilities $\frac{\lambda_{m}^{*}(t_{k}|H_{t_{k}})}{\lambda_{g}^{*}(t_{k}|H_{t_{k}})}$, *m* = {1, …, *M*−1}
    - set dN^*^_m_*k*__(*t*_*k*_) = 1 and dN^*^_m_(*t*_*k*_) = 0 for all *m* ≠ *m*_*k*_
  - else, set dN^*^_m_(*t*_*k*_) = 0 for all *m* ∈ {1, …, *M* − 1}
  - *dN*(*t*_*k*_) is obtained from dN^*^(*t*_*k*_) as in Supplemental Material 1
  - *k* = *k* + 1.

An alternative form of Algorithm 2 is given in Supplemental Material 1. We can also simulate data from an SEMPP model using an algorithm based on the univariate time-rescaling theorem (Brown et al., 2002) as follows:

**Algorithm 3 (Time-rescaling):** Given an interval (0, *T*]

1. Set *t*_0_ = 0 and ℓ = 1.
2. Draw *u*_ℓ_ from the uniform distribution on (0,1).
3. Find *t*_ℓ_ as the solution to: $\text{log}(u_{\ell })=\int_{t_{\ell -1}}^{t_{\ell }}\lambda_{g}^{*}(t|H_{t})dt$.
4. If *t*_ℓ_ > *T*, then stop, else
5. Draw *m*_ℓ_ from the (*M*−1)-dimensional multinomial distribution with probabilities $\frac{\lambda_{m}^{*}(t_{\ell }|H_{t_{\ell }})}{\lambda_{g}^{*}(t_{\ell }|H_{t_{\ell }})}$, *m* = {1, …, *M*−1}.
6. set dN^*^_m_ℓ__(*t*_ℓ_) = 1 and dN^*^_m_(*t*_ℓ_) = 0 for all *m* ≠ *m*_ℓ_.
7. *dN*(*t*_ℓ_) is obtained from dN^*^(*t*_ℓ_) as in Supplemental Material 1.
8. ℓ = ℓ + 1.
9. Go back to 2.

### Data analysis

To illustrate our model, we analyze simultaneously-recorded spiking activity data from pairs of neurons in the rat thalamus. The experiments were previously described in detail in Temereanca et al. (2008).

#### Experiment

Simultaneous single unit activity from pairs of thalamic neurons was recorded with two electrodes placed in the same electro physiologically-identified barreloid of the rat ventral posteromedial nucleus. For the neurons analyzed here, spiking activity was recorded from the pairs in response to whisker deflections at 50 mm/s administered at 8 Hz for a period of 2000 ms. A delay period of 500 ms preceded and followed each stimulus period. For each neuronal pair, the responses were recorded across 50 trials. We divided the 50 trials into a training set and a test set by randomly choosing 1 of every sequence of 3 trials and assigning it to the training set (17 trials). The remaining trials were assigned to the test set (33 trials).

**Figure 1:**
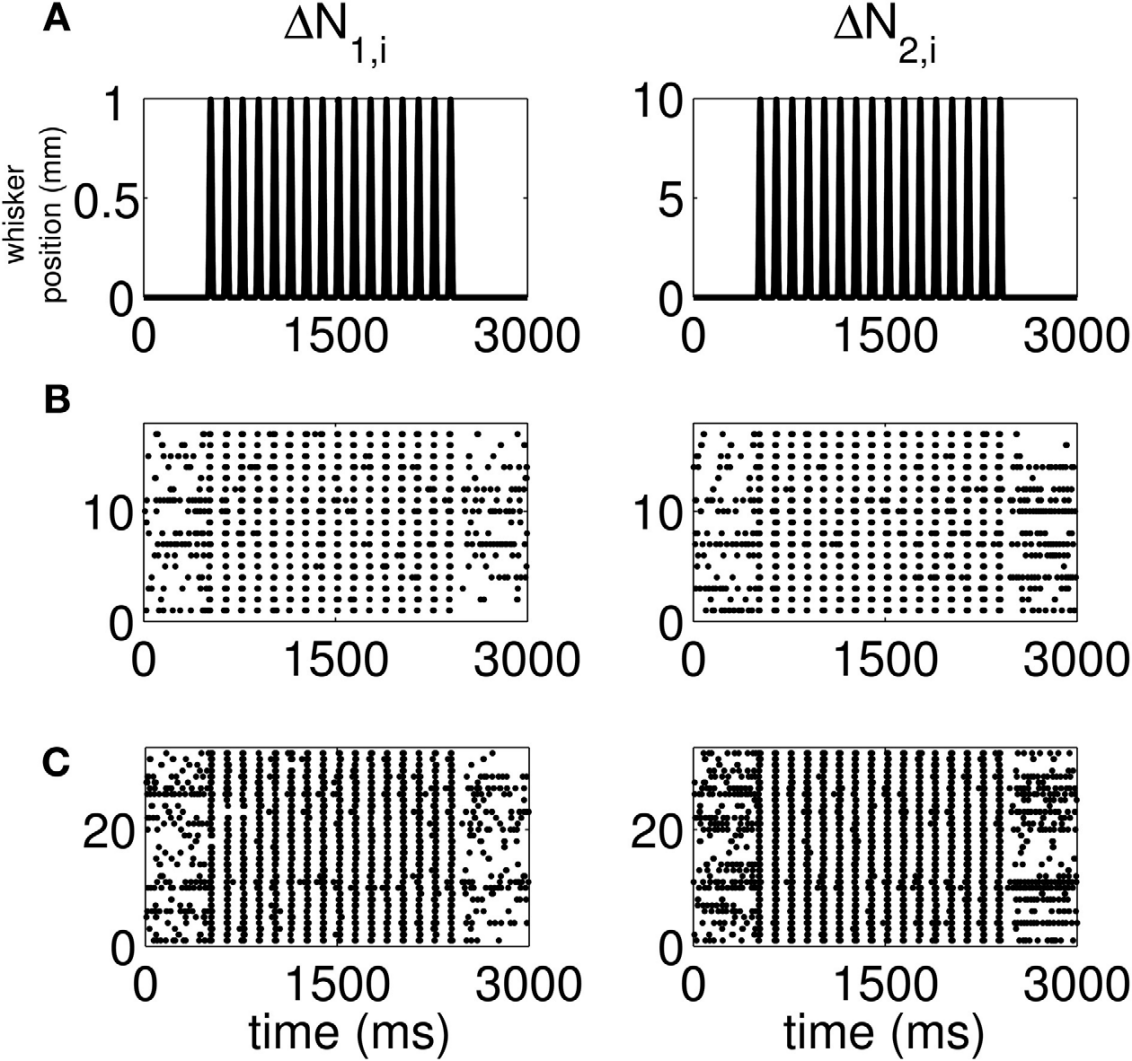
**Standard raster plots of the spiking activity of each neuron in a representative pair in response to a periodic whisker deflection of velocity *v* = 50 mm/s. (A)** Stimulus: periodic whisker deflection, **(B)** 17 trials of training data, **(C)** 33 trials of test data. The standard raster plots show that the stimulus induces strong modulation of the neural spiking in both the training and in the test sets. These standard raster plots do not clearly show the effect of the stimulus on joint spiking.

**Figure 2:**
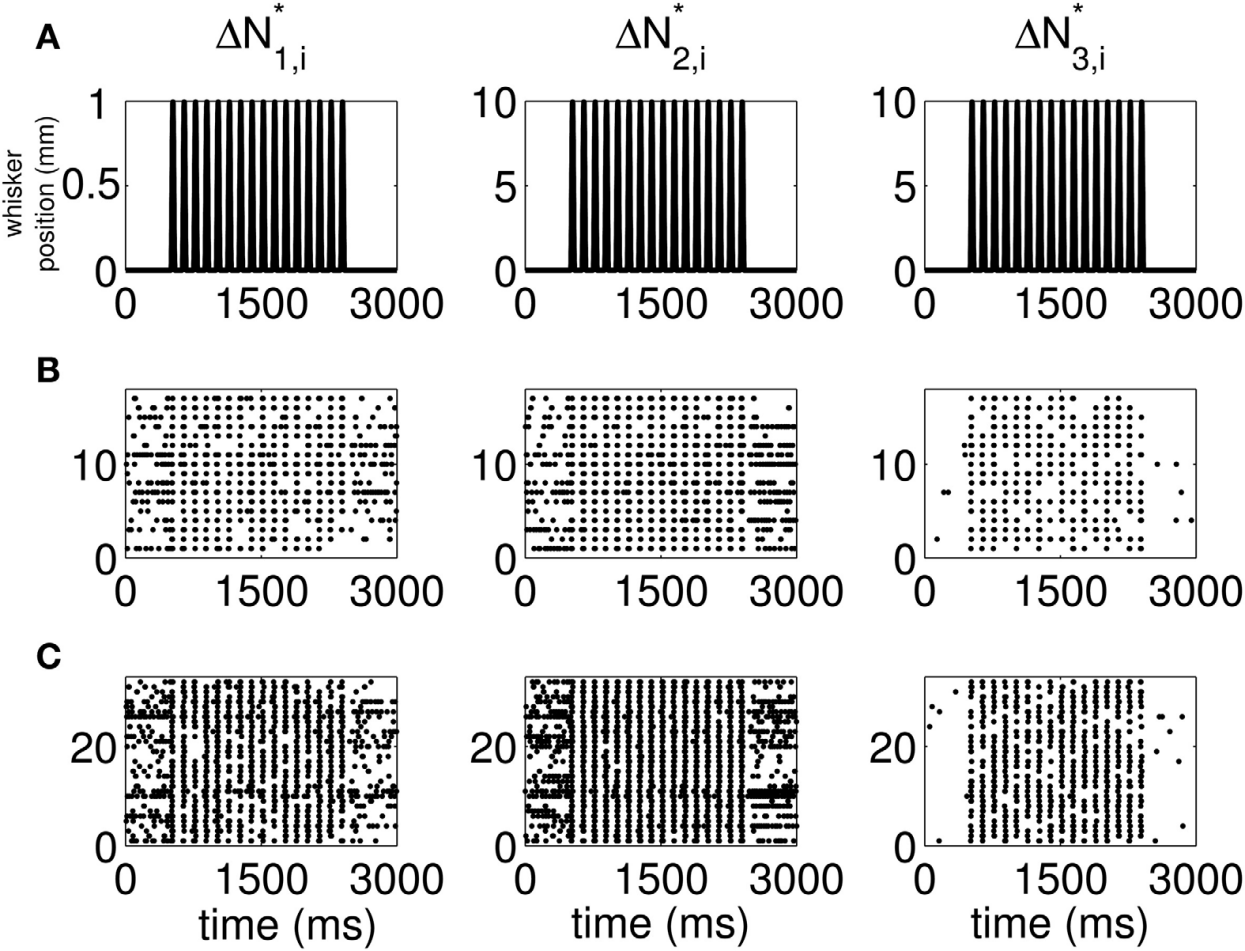
**New raster plots of non-simultaneous (“10” and “01,” Columns 1 and 2) and simultaneous (“11,” Column 3) spiking events for the neuron pair in Figure 1.** Each column corresponds to one of the components of ΔN^*^. **(A)** Stimulus, **(B)** 17 trials of training data, **(C)** 33 trials of test data. The new raster plots of the three components show clearly the effects of the stimulus on non-simultaneous and simultaneous spiking activity. The ΔN^*^_3,*i*_ component of ΔN^*^ shows that the joint spiking activity of the two neurons in response to the stimulus is pronounced.

The standard raster plots show that the stimulus (Figure 1A) induces strong modulation of the neural spiking in the training set (Figure 1B) and in the test set (Figure 1C). Our approach suggests an alternative raster plot which shows more clearly the simultaneous spiking activity of the pairs. Our new raster plots of the three components of ΔN^*^ (Figure 2) show more clearly the effects of the stimulus (Figure 2A) on the training set (Figure 2B) and the test set (Figure 2C). The ΔN^*^_3,*i*_ component of ΔN^*^ shows the joint spiking activity of the two neurons in response to the stimulus. This is the component that analyses based on the Jacod likelihood point-process models would simply ignore. For our analyses, we have *C* = 2, *M* = 4 and Δ = 1 ms.

#### Statistical model

We assume that the data are a sample from a bivariate SEMPP, whose discrete-time likelihood can be written as a product of conditional four-nomial trials. If we let

(12) $$\begin{array}{l} \text{log}\left[\frac{\lambda_{m}^{*}[i|H_{i}]\Delta }{1-\lambda_{g}^{*}[i|H_{i}]\Delta }\right]=\beta_{m,0}+\sum_{j=0}^{J-1}\beta_{m,j}^{\left(0\right)}s_{i-j}\\ \;+\sum_{c=1}^{2}\sum_{k=1}^{K_{c}}\beta_{m,k}^{\left(c\right)}\Delta N_{c,i-k} \end{array}$$

then our model becomes an mGLM with four-nomial observations and logit link (Fahrmeir and Tutz, 2001). The model expresses the log odds of each outcome with respect to Δ*N*_*g*,*i*_ = (0, …, 0)′ outcome as the convolution of the stimulus *s* with a finite length kernel {β^(0)^_*m*,*j*_}^*J* − 1^_*j* = 0_, and the history of Δ*N*_1_ and Δ*N*_2_, respectively with finite length kernels {β^(1)^_*m*,*k*_}^*K*_1_^_*k* = 1_ and {β^(2)^_*m*,*k*_}^*K*_2_^_*k* = 1_. Estimation is performed by maximizing the discrete-time likelihood [Equation (9) re-arranged as in Equation (S28), Supplemental Material 2] of the data under the model in Equation (12). We select *J*, *K*_1_ and *K*_2_ using Akaike's information criterion (AIC) and assess goodness-of-fit using the time-rescaling theorem to construct KS plots (Brown et al., 2002). In Supplemental Material 1, we state the multivariate time-rescaling theorem (Proposition 7.4.VI in Daley and Vere-Jones, 2003) and describe how to construct the KS plots we use to assess goodness of fit.

#### Assessing independence

It is not hard to show that the components of a bivariate Bernoulli random vector are independent if and only if they are uncorrelated. In each discrete-time bin, the model of Equation (12) results in an estimate of a *joint* pmf, conditioned on history. Therefore, we can assess the time-varying dependence between the neurons in a pair using the (conditional) covariance in each time bin.

#### Algorithms for fitting GLMs to SEMPP data

The goal of algorithms for fitting a model to discrete-time SEMPP data is to estimate the time and/history-dependent CIFs of the components of the SEMPP model. Conventional approaches are approximate ones which, given history, *separately* estimate the CIFs of each of the components of the SEMPP model. In the mGLM framework, one *jointly* estimates the CIFs of the components of the SEMPP model, thereby preserving the true discrete-time nature of the data.

More specifically, conventional approaches (Chornoboy et al., 1988; Okatan et al., 2005) fit a GLM to the discrete-time SEMPP data by first discretizing the continuous likelihood of Equation (10), to obtain the approximate discrete-time likelihood of Equation (S15), Supplemental Material 1. In effect, these approaches are similar to that of Berman and Turner (1992) in the univariate case. The resulting approximate discrete-time likelihood is the product of discrete-time univariate point process likelihoods. Then, using algorithms available in standard scientific computing packages, *separate* univariate Poisson GLMs are fit to the data.

In contrast, our mGLM algorithm uses the exact discrete-time likelihood of Equation (9), thus treating the multivariate SEMPP data as samples of conditional multinomial trials. The mGLM algorithm *jointly* fits multiple components to the data. This allows us to simultaneously estimate the time and/or history-dependent CIFs of the components of the SEMPP. This approach is different from conventional ones which assume that the components of the SEMPP are independent conditioned on history. The impact of independence conditoned on history is that it allows for fitting of *separate* univariate point-process models to the components of the SEMPP. The degree of conditional independence depends on how much history is allowed in process (e.g., 10, 20, 50).

Both approaches perform maximum likelihood estimation using Newton's method. One can recover the conventional approach of fitting separate GLMs to the components of the discrete-time SEMPP data by assuming that certain terms in the Hessian of the exact discrete-time likelihood are *o*(Δ). The Hessian of the exact discrete-time likelihood [after substituting Supplemental Material 2 Equation (S30) in (S28)] involves the multinomial covariance matrix, the entries of which are of the form λ^*^_m_Δδ_*m*,*m*′_ − λ^*^_m_Δ · λ^*^_m′_Δ, where δ_*m*,*m*′_ = 1, if *m* = *m*′ and 0 otherwise, *m*,*m*′ = 1, …, *M* − 1. If we assume that the λ^*^_m_Δ · λ^*^_m′_Δ component is *o*(Δ), the Hessian of the mGLM becomes block-diagonal. Then, each block corresponds to the Hessian matrix for the parameters of *one* of the components of the SEMPP. The block-diagonal structure of the Hessian implies that the parameters for each component can be estimated separately, as conventional approaches do.

This approximate equivalence between the multinomial GLM and multiple separate univariate Poisson GLMs extends the equivalence developed in Truccolo et al. (2005) between univariate Poisson and Bernoulli GLMs.

As we'll see below, conventional approaches are simpler and typically faster than the mGLM, due to their parallel nature. However, they require the CIF of the ground process to be uniformly small. This is an assumption that may be plausible in some cases but is hard to justify for all data from neurophysiology experiments, particularly those that use explicit stimuli such as the data from the whisking experiment (Temereanca et al., 2008), a small subset of which we analyze below.

#### An alternate SEMPP model and algorithm

Kass et al. fit separate GLMs to each component of the discrete-time representation of *N*(*t*). Then, they quantify the amount of excess simultaneous events that is observed beyond what is expected under independence (Kass et al., 2011). The mGLM algorithm results in a time-varying assessment of dependence, conditioned on history. In the case of Kass et al., the dependence structure is summarized using several scalar statistics. In that sense, the mGLM algorithm results in a finer characterization of the dependence structure of the components of the SEMPP. In the case of a bivariate SEMPP, this dependence structure is fully-summarized by the covariance between its components. The mGLM algorithm gives a time-varying estimate of this covariance as function of the relevant covariates, as well as history.

## Results

### Stimulus-induced increases in joint firing

To select the optimal model order of our model (Equation 12), we considered values for *J*, *K*_1_, and *K*_2_ ranging from 2 to 50 ms, in 1 ms increments. We used the results of preliminary GLM analyses on each neuron separately to reduce the dimension of the search space. The optimal orders obtained were *J* = 5, *K*_1_ = 37, and *K*_2_ = 14.

We found that reducing *J* to a value as low as *J* = 2 did not affect the goodness-of-fit, as measured by the number of points outside of the 95% confidence bounds in the KS plots. Therefore, the results we report here are for *J* = 2, *K*_1_ = 37, and *K*_2_ = 14.

**Figure 3:**
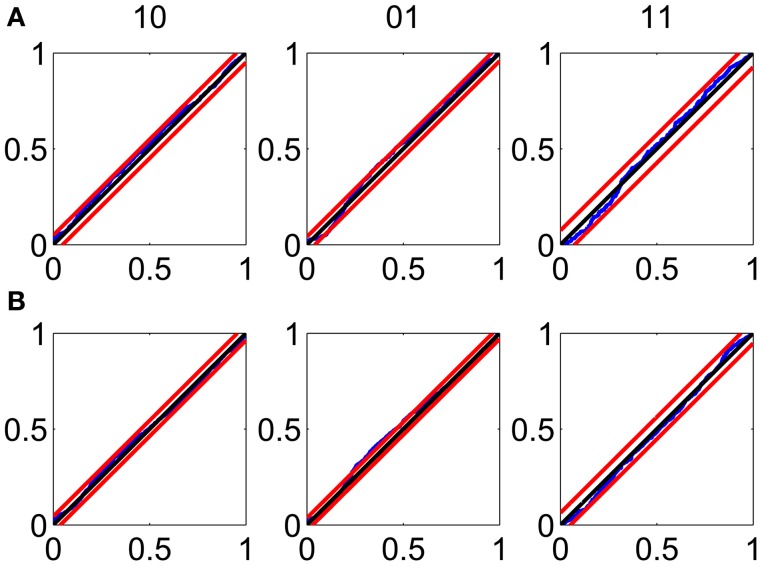
**Goodness-of-fit assessment by KS plots based on the time-rescaling theorem. (A)** Time-rescaling performance on the training data. **(B)** Time-rescaling performance on the test data. In both cases, the parallel red lines correspond to the 95% confidence bounds. The KS plots show that the model fits both the training and test data well. The good KS performance on each of the components of ΔN^*^ demonstrates the model's accurate description of the *joint* process. The performance on the test data demonstrates the strong predictive power of the model.

The KS plots show that the model fits both the training (Figure 3A) and test data (Figure 3B) well. The good KS performance on each of the components of ΔN^*^ demonstrates the model's accurate description of the *joint* process. The performance on the test data demonstrates the strong predictive power of the model.

**Figure 4:**
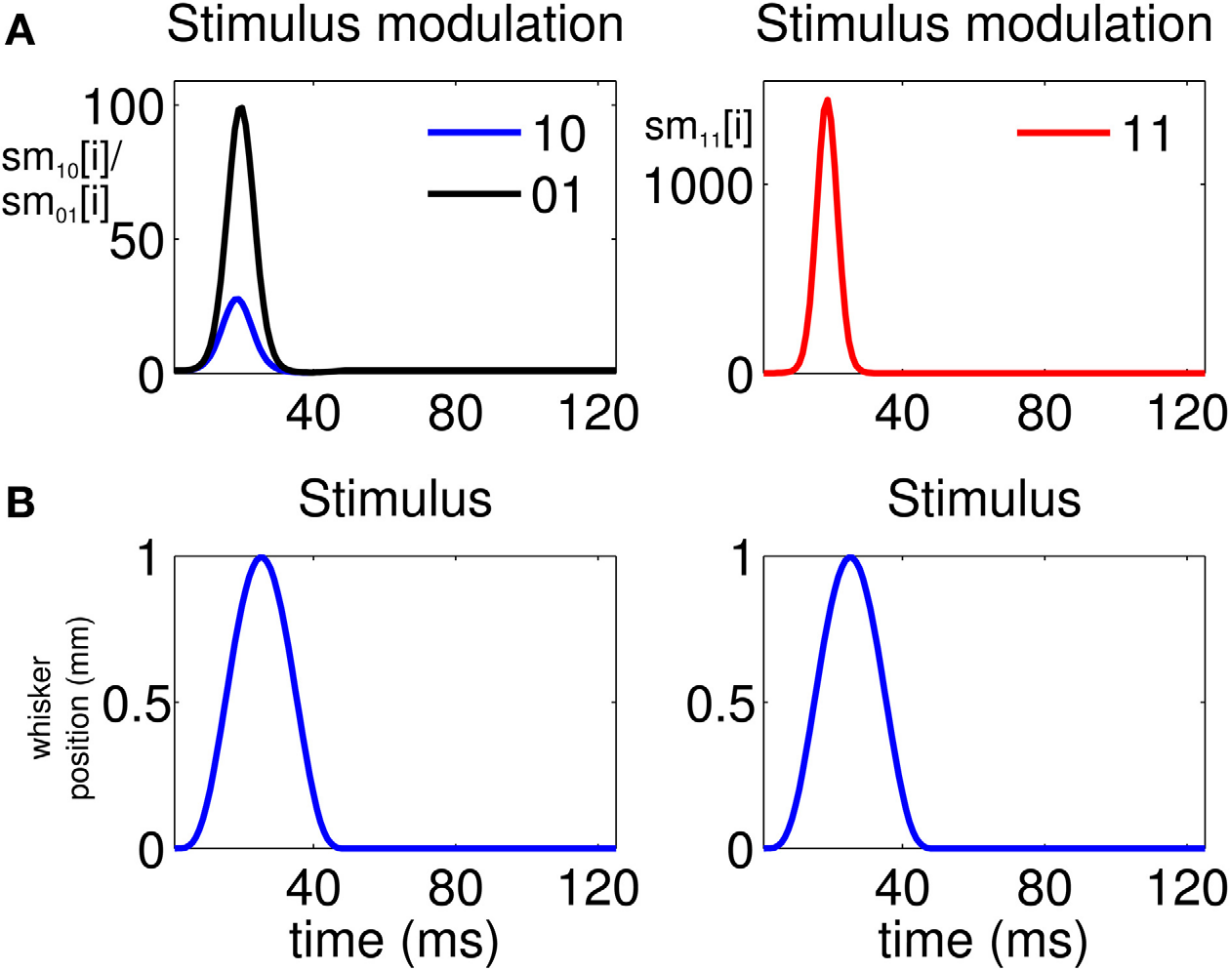
**Stimulus modulation of non-simultaneous (“10” and “01”) and simultaneous (“11”) events, over a single stimulus period.** The stimulus modulation of an event is the amount by which the stimulus increases the probability of that event. **(A)** Left: Stimulus modulation of the non-simultaneous events, $\left(\text{exp}\left\{\sum_{j=0}^{J-1}\beta_{m,j}^{\left(0\right)}s_{i-j}\right\},m=1,2\right)$. Right: Stimulus modulation of the simultaneous event, $\left(\text{exp}\left\{\sum_{j=0}^{J-1}\beta_{3,j}^{\left(0\right)}s_{i-j}\right\}\right)$. **(B)** Stimulus over a single period. The figure shows that the maximum amount by which the stimulus increases the probability of joint spiking is one order of magnitude greater than that by which the stimulus increases the probability of non-simultaneous spiking. Thus, simultaneous spiking can be attributed for the most part to the administration of the stimulus.

The analysis indicates that there is a high propensity for simultaneous firing due to the stimulus. This is reflected by the stimulus modulation of the simultaneous-spiking event (“11”) (Figure 4A, Column 2), which is defined by

(13) $$sm_{11}[i]=\text{exp}\left\{\sum_{j=0}^{J-1}\beta_{3,j}^{\left(0\right)}s_{i-j}\right\}$$

This is a unitless quantity which represents, at a ms time scale, the amount by which the stimulus increases the probability of a simultaneous event. Similarly, we can define the quantities

(14) $$sm_{01}[i]=\text{exp}\left\{\sum_{j=0}^{J-1}\beta_{2,j}^{\left(0\right)}s_{i-j}\right\}$$

and

(15) $$sm_{10}[i]=\text{exp}\left\{\sum_{j=0}^{J-1}\beta_{1,j}^{\left(0\right)}s_{i-j}\right\},$$

which represent the stimulus modulation of the “01” and “10” events, respectively. As Figure 4A, Column 1 suggests, there is also a modulation of the spiking activity in response to the stimulus for the non-simultaneous events (“10” and “01”). However, the maximum stimulus effect for the simultaneous spiking (≈1200) is one order of magnitude stronger than the maximum effect for the non-simultaneous spiking (≈90).

From our analysis, we can compute a time-varying estimate of the CIFs for each of the 17 trials used for training, and hence of the covariance between the neurons for each of these trials. For a bivariate binary process, the covariance fully summarizes the dependence between its components. For visualization purposes, we average the CIF estimates across trials and use these averages to obtain a trial-averaged covariance estimate. Using the trial-averaged covariance estimate, as well as the trial-averaged CIFs, we compute a trial-averaged correlation coefficient estimate as follows:

(16) $$\rho [i]=\frac{\lambda_{3}^{*}[i|H_{i}]\Delta -\lambda_{1}[i|H_{i}]\Delta \cdot \lambda_{2}[i|H_{i}]\Delta }{\sqrt{\lambda_{1}[i|H_{i}]\Delta (1-\lambda_{1}[i|H_{i}]\Delta )\lambda_{2}[i|H_{i}]\Delta (1-\lambda_{2}[i|H_{i}]\Delta )}}.$$

**Figure 5:**
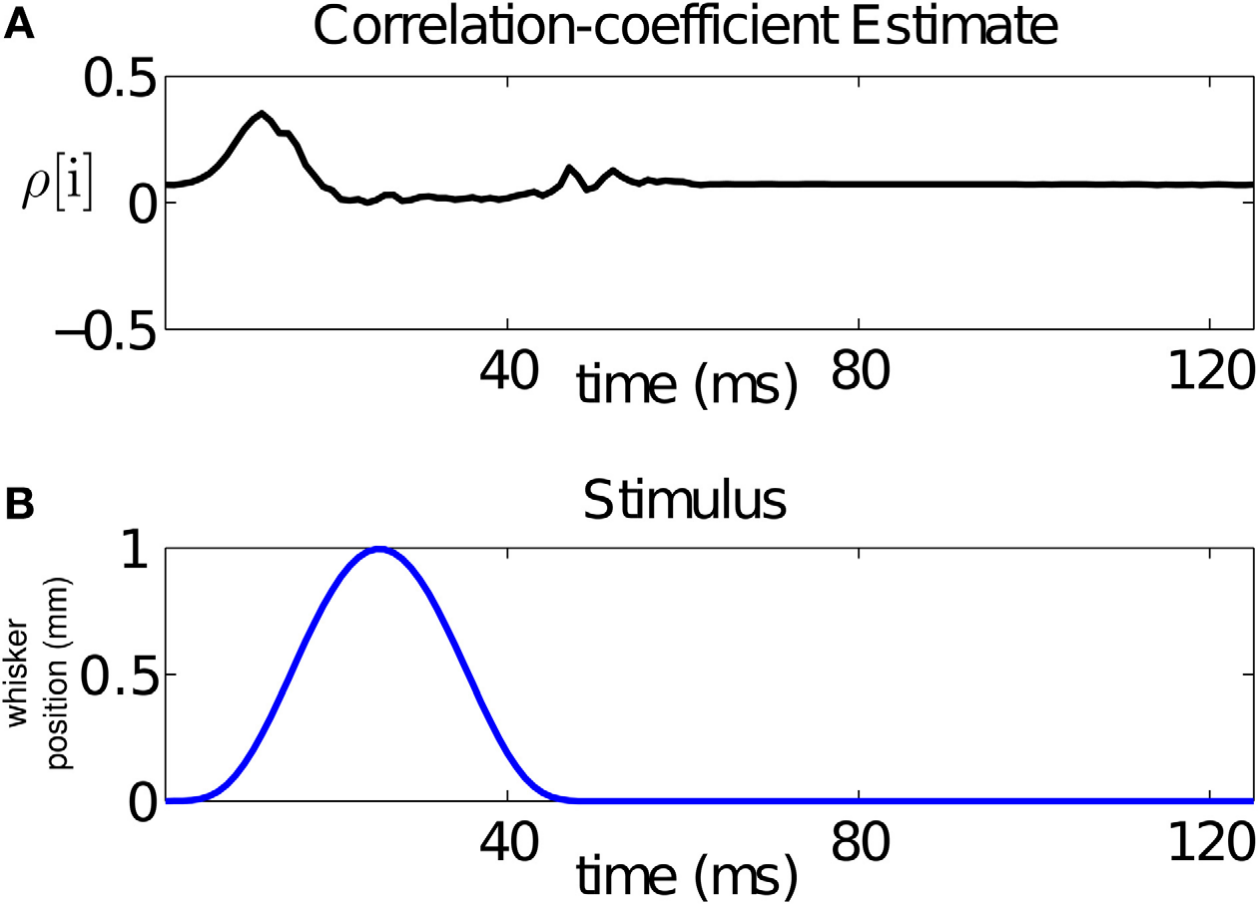
**Time-varying assessment of the correlation between the pair of neurons in Figure 1. (A)** Estimate of the correlation coefficient, **(B)** stimulus over a single period. The figure demonstrates that the stimulus changes the correlation structure, hence the dependence, between the neurons in the pair at the ms time scale. For this pair, the rising cycle of the stimulus increases the correlation beyond baseline and then decreases it, while the falling cycle makes the uncorrelated, before returning to baseline.

Since the stimulus is periodic in the interval between 500 and 2500 ms (with period 125 ms), we average our estimate of the across-trial correlation coefficient over the 16 stimulus cycles. The result is displayed in Figure 5A. The figure demonstrates that the stimulus changes the correlation structure, hence the dependence, between the neurons in the pair at the ms time scale. In particular, changes in the correlation coefficient mirror changes in the stimulus. For this pair, the rising cycle of the stimulus increases the correlation beyond baseline and then decreases it, while the falling cycle makes the neurons uncorrelated, before returning to baseline.

Despite the strong stimulus modulation of simultaneous firing, there is a smaller number of simultaneous occurrences than non-simultaneous occurrences (Figure 2). The intrinsic dynamics of Neuron 1 and Neuron 2 (Figure S8C, Columns 2 and 3) limit their high propensity to fire simultaneously. Indeed, in these supplemental figures (available in Supplemental Material 3 on the Frontiers website), the first few coefficients corresponding to the effect of Neurons 1 and 2 on the probability of simultaneous firing are negative for *both* neurons. This means that if both neurons have just fired, their probability of simultaneous firing within the next 1–3 ms decreases significantly. On the other hand, for non-simultaneous events, *only one* of the set of first few coefficients mentioned above is negative. This explains why we observe a greater number of non-simultaneous than simultaneous occurrences.

Although some of the 95% confidence intervals for the individual parameters (Figure S8) overlap with zero, these small coefficients contribute significantly to the descriptive (Figure 3A) and predictive (Figure 3B) power of the model. Results of analysis of a different pair of neurons are in Figures S9–S14 (Supplemental Material 3), which can be found on the Frontiers website.

### Simulated neural spiking data

**Figure 6:**
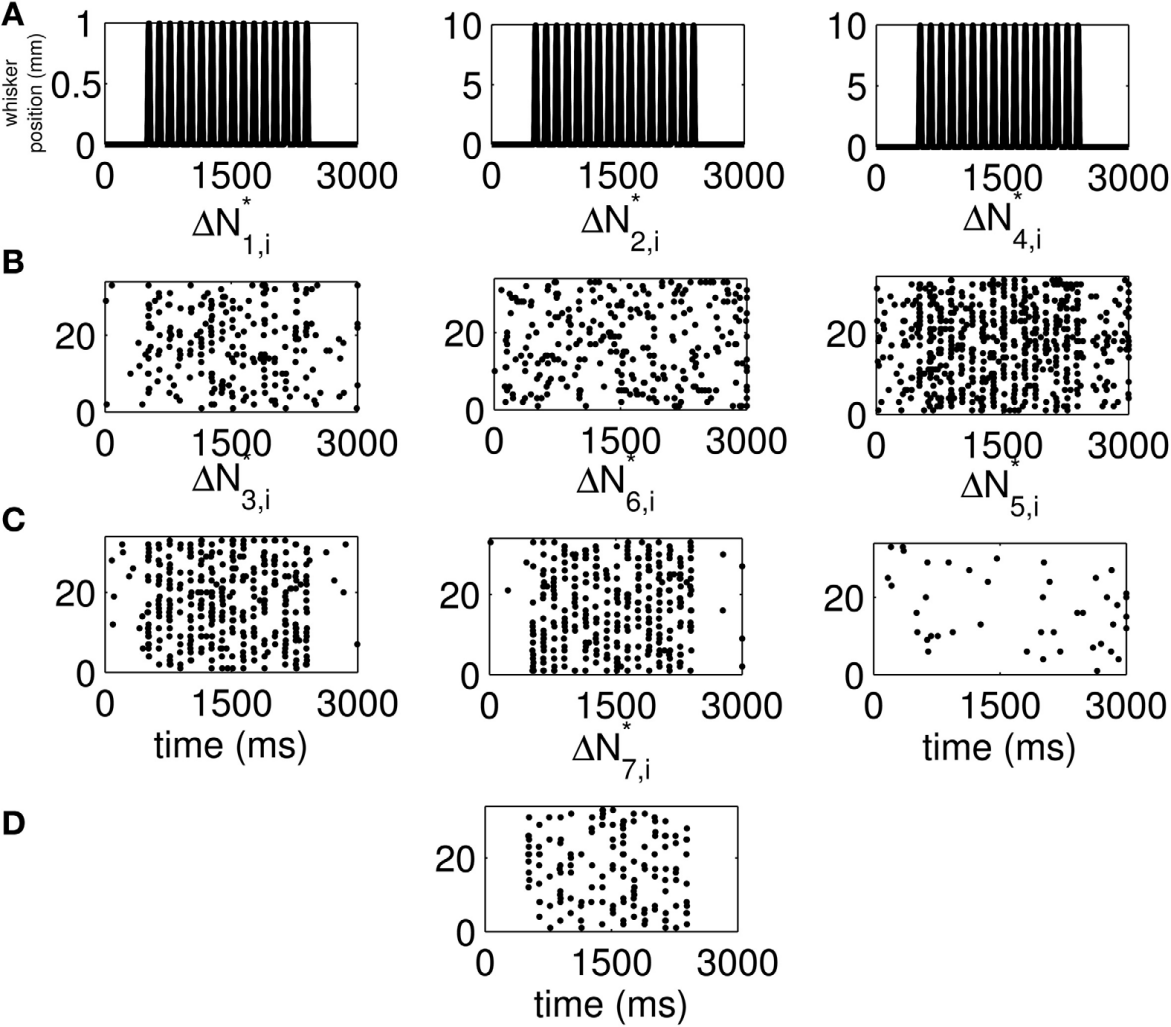
**New raster plots of non-simultaneous (“100,” “010,” and “001”) and simultaneous (“110,” “011,” “101,” and “111”) spiking events for the three simulated neurons of in Figure S15. (A)** Stimulus (same as in Figures 1A, 2A). **(B)** Non-simultaneous events, from left to right, “100,” “010,” and “001.” **(C)** Simultaneous events from pairs of neurons, from left to right, “110,” “011,” and “101.” **(D)** Simultaneous event from the three neurons (“111”). The new raster plots of the three components show clearly the effects of the stimulus on non-simultaneous and simultaneous spiking. The ΔN^*^_4,*i*_ and ΔN^*^_5,*i*_ components of ΔN^*^ show that the joint spiking activity of the pairs consisting of Neurons 1 and 2 on the one hand, and Neurons 2 and 3 on the other hand is pronounced. The ΔN^*^_7,*i*_ component of ΔN^*^ shows that the joint spiking activity of the three neurons is also pronounced. The information in these raster plots about the joint spiking activity of neurons could not be gathered from Figure S15.

We use the time-rescaling algorithm (Algorithm 3) to simulate simultaneous spiking activity from three thalamic neurons in response to periodic whisker deflections of velocity 50 mm/s. We model the CIFs of the neurons as in Equation (12) to simulate 33 trials of the experiment described above. For these simulations, we chose *J* = 2, *K*_1_ = 2, *K*_2_ = 2, and *K*_3_ = 2. Figure S15 shows the standard raster plots of the simulated data. There is strong modulation of the activity of each of the neurons by the stimulus. Figure 6 shows the raster plots of each of the seven disjoint components of ΔN^*^. As the figure indicates, the parameters of the model were chosen so that the stimulus strongly modulates simultaneous occurrences from the pairs Neuron 1 and Neuron 2, Neuron 2 and Neuron 3, as well as simultaneous occurrences from the triple.

### Comparing mGLM and separate univariate GLMs

We used the data from (Figure 1) to compare the mGLM algorithm to the approximate algorithm which fits separate univariate Bernoulli GLMs to each of the components of the disjoint representation (Figure 2).

The mGLM, as well as algorithms for fitting univariate Bernoulli GLM, can be implemented using Newton's method. Each Newton step corresponds to a high-structured linear system, which is typically solved using the QR-decomposition. Our experience with neural data sets shows that using linear conjugate gradient (CG) instead can significantly speed up computation (Komarek and Moor, 2005; Ba, 2011). This can be mainly attributed to the sparse patterns of design matrices that arise from neural data sets. Therefore, our comparisons use CG-based implementations of the algorithms for fitting the mGLM and the univariate Bernoulli GLM. The CG algorithm requires a tolerance to which the linear system is solved, as well as a maximum number of iterations to perform. We set the tolerance to 10^−10^, and the number of CG iterations to the maximum possible, which is the dimension of the linear system. Each CG iteration is initialized using the parameters of the previous Newton update. We use the same values of *J*, *K*_1_, and *K*_2_ as in **Data Analysis** to fit the models.

**Figure 7:**
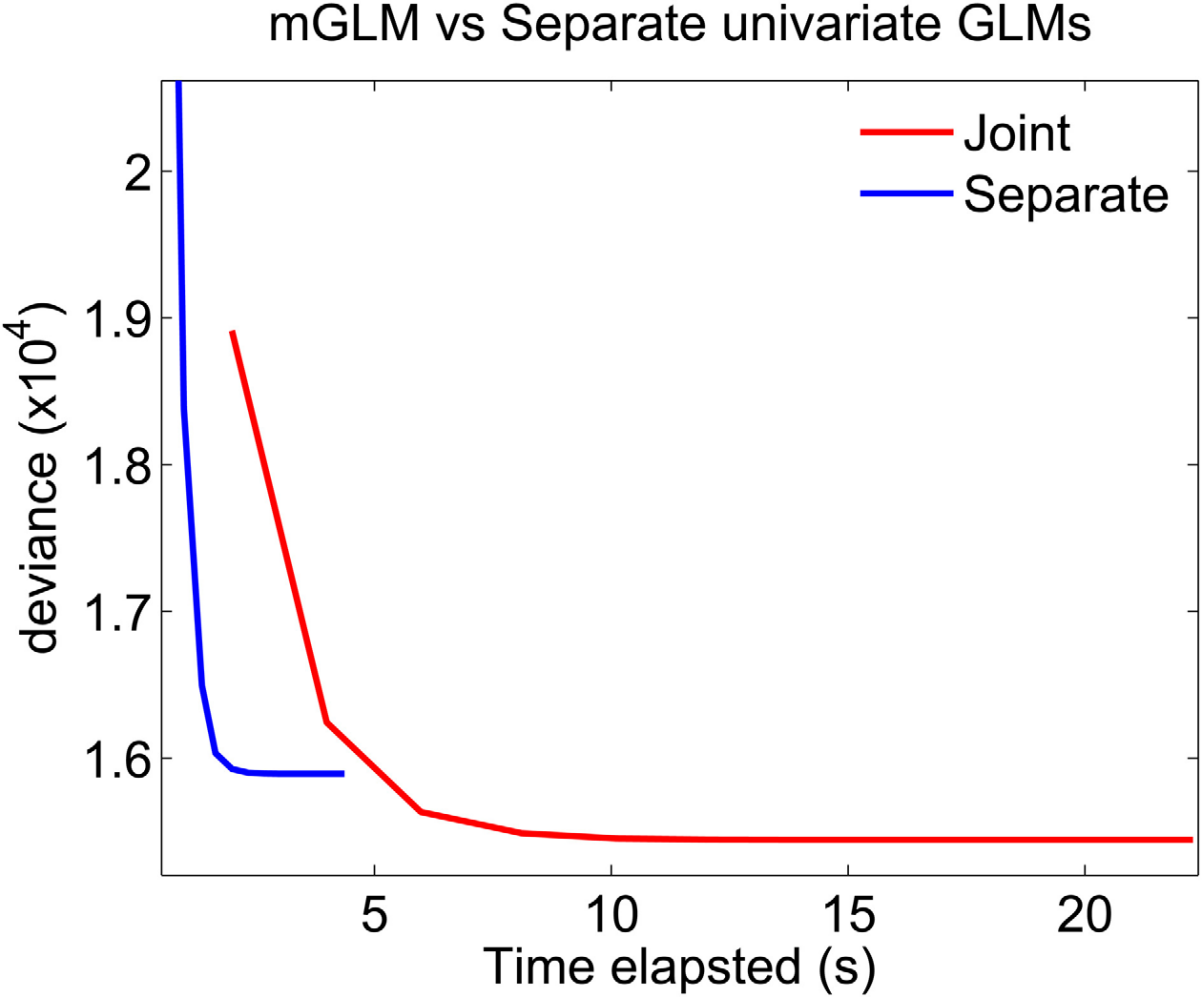
**Fitting mGLM model as opposed to Separate Univariate GLMs.** For the example in **Data Analysis**, the mGLM algorithm achieves a smaller value of the deviance (larger value of the likelihood), but is slower than the algorithm which fits separate univariate GLMs. Comparison of the coefficient estimates would reveal that the latter algorithm underestimates the coefficients corresponding to the simultaneous “11” event.

Figure 7 plots the deviance of each of the algorithms as a function of time elapsed from the beginning of the algorithm to the end of a Newton iteration. For GLMs, the deviance generalizes the idea of mean-squared error for linear models: minimizing the deviance is equivalent to maximizing the likelihood. The algorithms which fits separate GLMs is parallel in nature. Therefore, to compute the time elapsed until the end of the *k*th Newton iteration, we use the maximum (across all separate components) of the time elapsed until the end of each component's *k*th iteration. The figure shows that the mGLM algorithm achieves a smaller value of the deviance (larger value of the likelihood), but is slower to converge. That is, the mGLM required ≈5 s to reach the value of the deviance that the algorithm which fits separate GLMs converged to in ≈2 s.

## Discussion

### mGLM: a versatile paradigm for encoding analyses of ensemble neural activity

We have presented a new algorithmic framework for the analysis of ensemble neural spiking activity based on the joint probability density of a MPP with simultaneous events. We have developed an equivalent MkPP representation of this joint density which, together with the previously-described continuous and discrete-time representations (Solo, 2007), provide a principled way to conduct model-based data analyses and simulation studies of ensemble neural activity that offer several advantages over current analyses approaches.

#### Likelihood-based analyses

The discrete-time SEMPP representation provides an efficient means of performing likelihood analyses of multiple simultaneously-recorded neurons using an mGLM algorithm for model fitting (Fahrmeir and Tutz, 2001) and the disjoint representation (Equation 10) (Solo, 2007) allows us to assess goodness-of-fit using the time-rescaling theorem. The mGLM algorithm is a multivariate extension of the GLM algorithm used to fit univariate point process models to single neural spike trains (Paninski, 2004; Truccolo et al., 2005). Unlike existing approaches to fitting a GLM to SEMPP data, the mGLM jointly estimates the CIFs of the component of the SEMPP, and achieves a larger value of the likelihood (Figure 7). The algorithm which fits separate components achieves coefficient estimates which are very close to the ones of the mGLM algorithm, for the non-simultaneous components (“10” and “01”), but less so for the simultaneous component (“11” event) (data not shown). Our intuition tells us that the coefficients of the simultaneous “11” event are underestimated due to few number of events (Figure 2), coupled with the fact that the separate fitting procedure, unlike the mGLM algorithm, does not use second-order correlation information between its components.

#### Assessing goodness-of-fit

The goodness-of-fit analysis (Figure 3) uses the MPP time-rescaling theorem (Daley and Vere-Jones, 2003; Vere-Jones and Schoenberg, 2004) and is a multivariate extension of the one-dimensional time-rescaling theorem used to assess goodness-of-fit for univariate point process models (Brown et al., 2002). Once adequate goodness-of-fit is established, our inference framework is likelihood-based. Therefore, it uses the Fisher information to compute parameter standard errors and confidence intervals (Figures S8, S14), and established procedures such as AIC and BIC to perform model selection. Our approach also carries all the optimality properties of the likelihood framework.

#### Analyzing an arbitrary number of neurons

Our paradigm applies to multiple neurons and is therefore more general than the common bivariate histogram methods (Gerstein and Perkel, 1969; Brillinger et al., 1976; Brody, 1999). It also obviates the stationarity assumption required by histogram methods (Gerstein and Perkel, 1969; Brillinger et al., 1976; Brody, 1999). Unlike the Jacod likelihood (Chornoboy et al., 1988; Karr, 1991; Okatan et al., 2005), the SEMPP model allows simultaneous events at an arbitrarily small time-scale. Although we only applied our techniques to simultaneous recordings from pairs of neurons, our approach characterizes in each small time interval of length Δ the probability of all 2^*C*^−1 patterns of spiking activity from *C* neurons. For this reason, our approach offers a key improvement over pattern analysis algorithms (Grün et al., 2002; Gütig et al., 2002; Pipa and Grün, 2002; Grün, 2009) because it analyzes in a likelihood framework all of the patterns defined by the recording resolution Δ.

#### Simulating ensemble neural activity

The MkPP representation also gave a new thinning and a new time-rescaling algorithm for simulating simultaneous neural spiking activity in continuous time (Figures 6, S15). This makes it possible to use the same modeling approach for data analysis and for simulation studies. The MkPP representation follows from the observation that, in any small time interval Δ, the SEMPP is a multinomial model with 2^*C*^ possible outcomes. A multinomial model with 2^*C*^ outcomes can be written as the product of a binomial probability model and a conditional multinomial probability model with 2^*C*^−1 outcomes. The binomial probability model defines the ground process and the conditional multinomial process defines the marked process. The simulation algorithms from the MkPP representation (Algorithms 2 and 3) are simpler than one based on the time-rescaling theorem for MPPs (Algorithm 1), which uses the disjoint representation.

### Joint encoding of whisker motion by two pairs of thalamic neurons

Our analysis of the joint spiking activity of the pair of thalamic neurons in response to whisker stimulation reveals the types of new insights that could be learned from the SEMPP model. It suggests a new raster plot to assess simultaneity (Figure 2, Column 3). The key finding of our model analysis that is not apparent from this new raster plot is that the stimulus modulation of the joint spiking activity is more than one order of magnitude greater than its modulation of either of the unpaired spiking activity (Figure 4). These estimates of the stimulus modulation are model-based spike-triggered averages (Simoncelli et al., 2004). Our analysis also reveals that changes in the correlation structure, and hence the dependence structure, between the neurons in a pair mirror changes in the stimulus (Figures 5, S13). These brief analyses suggest that our approach could offer new insights into the importance of joint spiking activity for understanding representations of stimuli in ensemble neural spiking activity.

To our knowledge, these examples constitute the first practical demonstrations of the versatility of the SEMPP model for analysis and simulation of jointly-recorded neural spiking activity.

### Future directions and implications of the mGLM model

The new SEMPP model and analysis suggests several important future extensions of this work.

#### Implications of mGLM for joint encoding of whisker motion by pairs of thalamic neurons

The pair of neurons analyzed here, as well as the pair in Figures S9–S14, are from a set of 17 pairs of thalamic neurons simultaneously recorded during stimulation by three different whisker velocities (Temereanca et al., 2008). In a future report, we will present our findings on the analysis of the complete data set using the SEMPP model fit with the mGLM algorithm.

#### Applications of mGLM to assessment of functional connectivity

By substituting the SEMPP likelihood for the Jacod likelihood, our methods suggest a new approach to analyzing functional connectivity in neuronal ensembles (Okatan et al., 2005), that may give a more accurate assessment of the extent to which it is modulated by the intrinsic dynamics of the neurons and/or external stimuli (Truccolo et al., 2009).

#### Decoding analyses of sensory representations and prediction of whisker motion

Thalamic neurons use their firing patterns to form a representation of the sensory information conveyed by whisker deflections (Temereanca et al., 2008). We constructed a highly accurate model of this sensory representation by applying the mGLM to simultaneous recordings from pairs of thalamic neurons in response to periodic whisker deflections. Encoding models fit using mGLM are arguabyly superior to existing approaches because of their ability to capture the effect of stimuli (e.g., whisker motion) on the simultaneous-spiking activity of neurons. As witnessed by our analyses of the thalamic data, this effect can be quite substantial. In such scenarios, when the effect of sensory stimuli on simultaneous spiking is pronounced, using the mGLM as an encoding model can result in superior decoding performance. For the thalamic data analyzed here, we believe that the ability to predict whisker motion using an encoding model fit using mGLM, and a carefully-designed decoding algorithm using the SEMPP likelihood (Ba, 2011), will be superior compared to the case when one uses the convential GLM (Paninski, 2004; Truccolo et al., 2005) for encoding and the Jacod likelihood (Eden et al., 2004) for decoding. Indeed, our SEMPP model also suggests a new approach to designing point-process filters for ensemble neural spike train decoding and adaptive filtering studies of neural plasticity (Brown et al., 1998, 2001). These new algorithms based on the disjoint representation make explicit use of simultaneous events. The computational complexity of fitting the SEMPP model increases exponentially with the number of neurons. Therefore, the design of more efficient model-fitting algorithms must be an important focus of future work.

#### Simultaneous spiking at smaller time scales

The impact of using smaller time scales (Δ), rather than the conventional 1 ms time scale, is an important question that we hope to address in future work. The mGLM and conventioal GLM (Paninski, 2004; Truccolo et al., 2005) are based on discrete-time approximations of the continuous-time likelihood of an SEMPP and univariate point-process, respectively. In recent work (Citi et al., 2013), we have introduced a novel discrete-time approximation to the continuous-time likelihood of a point-process that is significantly more accurate than that used in the conventional GLM (Paninski, 2004; Truccolo et al., 2005). We have demonstrated that the approximation used in the conventional GLM can require time scales that are up to one order of magnituded smaller (10 μ s as opposed to 1 ms) to achieve the same goodness-of-fit as our new approximation (Citi et al., 2013). The improvement comes from explicitly accounting for the refractory period of neurons in the new approximation. It is not hard to extend this formulation to the mGLM. We believe this formulation provides a better ground for assessing the impact of smaller time scales on the mGLM analyses.

### Conflict of interest statement

The authors declare that the research was conducted in the absence of any commercial or financial relationships that could be construed as a potential conflict of interest.
