# Supplementary material for: Algorithms for the analysis of ensemble neural spiking activity using simultaneous-event multivariate point-process models
Source: Front Comput Neurosci. 2014 Feb 10;8:6. doi: 10.3389/fncom.2014.00006 (PMC3918645; doi:10.3389/fncom.2014.00006)
Supplement: Supplementary file 1 [file Presentation1.PDF]

Supplemental Material 1 for "Algorithms for the Analysis of Ensemble Neural Spiking Activity Using Simultaneous-Event Multivariate Point-Process Models" by Ba, Temereanca and Brown

I - Description of One-to-one Mapping from $N(t)$ to $N^*(t)$: We describe *one* possible explicit map from $N(t)$ to $N^*(t)$ in the arbitrary C-variate case.

From $N(t)$ to $N^*(t)$: For each $t \in (0, T]$, the vector $dN(t) = (dN_1(t), \cdots, dN_C(t))'$ of counting measure increments of $N(t)$ has entries either 0 or 1. Therefore, we can treat $dN(t)$ as a C-length binary number. We let $m_{dN(t)} = \sum_{c=1}^{C} dN_c(t)2^{c-1}$ be the decimal (base-10) representation of $dN(t)$: $m_{dN(t)} \in \{0, \cdots, 2^C - 1\}$.

Consider the 2^C-1-dimensional vector $dN^*(t) = (dN_1^*(t), \cdots, dN_{2^C-1}^*(t))'$. If $m_{dN(t)} = 0$, we let $dN^*(t) = (0, \cdots, 0)'$. Otherwise, we let $dN_m^*(t) = 1$ if $m = m_{dN(t)}$ and $dN_m(t) = 0$ otherwise. In this case, $dN^*(t)$ is an indicator vector for the event $dN(t)$ which occurs at t. If we let $N_m^*(t) = \int_0^t dN_m^*(u)$, then $N^*(t) = (N_1^*(t), \cdots, N_{2^C-1}^*(t))'$ becomes a multivariate point-process of disjoint events from $N(t)$.

From $N^*(t)$ to $N(t)$: For each $t \in (0, T]$, the vector $dN^*(t) = (dN_1^*(t), \cdots, dN_{2^C-1}^*(t))'$ is either $(0, \cdots, 0)'$ or an indicator vector. In the former case, we let $dN(t) = (0, \cdots, 0)'$. In the latter case, we would like to determine the event $dN(t)$ that $dN^*(t)$ is an indicator of. Let $m \in \{1, \cdots, 2^C - 1\}$ be the index of the non-zero entry of $dN^*(t)$ and $b_m = b_{m1}b_{m2}\cdots b_{mC}$ be the binary representation of m. If we let $dN(t) = (b_{mC}, \cdots, b_{m2}, b_{m1})'$, we obtain the event $dN(t)$ that $dN^*(t)$ is an indicator of. Letting $N_c(t) = \int_0^t dN_c(u)$, we recover the C-variate SEMPP $N(t) = (N_1(t), \cdots, N_2(t))'$.

II - Derivation of the Ground Intensity and the Mark Probability Mass Function (PMF): We need to specify (a) the intensity of the ground process (Eq. 7) and (b) the distribution of the marks (Eq. 8). By definition,

$$\lambda_g^*(t|H_t) = \lim_{\Delta \to 0} \frac{P[\Delta N_{g,t} = 1|H_t]}{\Delta}. \tag{S1}$$

$$P[\Delta N_{g,t} = 1|H_t] = P\Big[\bigcup_{m=1}^{M-1} \Delta N_{m,t}^* = 1|H_t\Big] \tag{S2}$$

$$= \sum_{m=1}^{M-1} P\big[\Delta N_{m,t}^* = 1|H_t\big] + o(\Delta) \tag{S3}$$

$$= \sum_{m=1}^{M-1} \lambda_m^*(t|H_t)\Delta + o(\Delta), \tag{S4}$$

where the second equality follows from the fact that the events $\{\Delta N^*_{m,t} = 1 \cap \Delta N^*_{k,t} = 1\}$ are disjoint for all (m, k) given full history (i.e. ΔN^*_t has no simultaneities). From here, it is not hard to see that

$$\lambda^*_g(t|H_t) = \sum_{m=1}^{M-1} \lambda^*_m(t|H_t). \tag{S5}$$

The mark PMF requires a little more work. We are seeking an expression for $P\big[dN^*_m(t) = 1|dN_g(t) = 1, H_t\big]$ in terms of the $\lambda^*_m(t|H_t)$'s.

$$P\big[dN^*_m(t) = 1|dN_g(t) = 1, H_t\big] = \lim_{\Delta\to 0} P\big[\Delta N^*_{m,t} = 1|\Delta N_{g,t} = 1, H_t\big] \tag{S6}$$

$$= \lim_{\Delta\to 0} \frac{P\big[\Delta N^*_{m,t} = 1|H_t\big]}{P\big[\Delta N_{g,t} = 1|H_t\big]} \tag{S7}$$

$$= \lim_{\Delta\to 0} \frac{\lambda^*_m(t|H_t)\Delta + o(\Delta)}{\lambda^*_g(t|H_t)\Delta + o(\Delta)} \tag{S8}$$

$$= \frac{\lambda^*_m(t|H_t)}{\lambda^*_g(t|H_t)}, \tag{S9}$$

$m = 1, \cdots, M-1$, so that the marks follow a multinomial distribution with probabilities given as above.

III - Discrete-time Notation: Choose I large and partition the interval $(0, T]$ into subintervals of width $\Delta = I^{-1}T$. In discrete-time $N_c(t)$ and $N^*_m(t)$ are respectively $N_{c,i} = N_c(i\Delta)$, $N^*_{m,i} = N^*_m(i\Delta)$ for $i = 1, \cdots, I$. Let $\Delta N_{c,i} = N_{c,i} - N_{c,i-1}$, and $\Delta N^*_{m,i} = N^*_{m,i} - N^*_{m,i-1}$. Letting $\Delta N_i = (\Delta N_{1,i}, \cdots, \Delta N_{C,i})'$, we choose I large enough so that $\Delta N_{c,i}$ is 0 or 1. Either $\Delta N^*_i = (\Delta N^*_{1,i}, \cdots, \Delta N^*_{M-1,i})'$ has one event in exactly one component or $\Delta N^*_i = (0, \cdots, 0)'$. Let $\Delta N^* = (\Delta N^*_1, \cdots, \Delta N^*_I)'$ be the $I \times M-1$ matrix of discretized outcomes for the observation interval $(0, T]$.

IV - Derivation of the Discrete-time Joint PDF of the Disjoint Process: To arrive at the discrete-time joint PDF of Eq. 9, we first need the PDF $P\big[\Delta N^*_i|H_i\big]$ in the ith interval. Conditioned on history, this PDF is given by

$$P\big[\Delta N^*_i|H_i\big] = \prod_{m=1}^{M-1} (\lambda^*_m[i|H_i]\Delta)^{\Delta N^*_{m,i}} \left(1 - \sum_{m=1}^{M-1} \lambda^*_m[i|H_i]\Delta\right)^{1-\sum_{m=1}^{M-1}\Delta N^*_{m,i}}, \tag{S10}$$

$$= \prod_{m=1}^{M-1} (\lambda^*_m[i|H_i]\Delta)^{\Delta N^*_{m,i}} \left(1 - \lambda^*_g[i|H_i]\Delta\right)^{1-\Delta N_{g,i}}, \tag{S11}$$

where $\Delta N_{g,i} = N_{g,i} - N_{g,i-1} = \sum_{m=1}^{M-1} \Delta N^*_{m,i}$, $N_{g,i} = N_g(i\Delta)$. The joint PDF of ΔN^* is

$$P\big[\Delta N^*\big] = \prod_{i=1}^{I} P\big[\Delta N^*_i|H_i\big] \tag{S12}$$

$$= \prod_{i=1}^{I}\prod_{m=1}^{M-1} (\lambda^*_m[i|H_i]\Delta)^{\Delta N^*_{m,i}} \left(1 - \lambda^*_g[i|H_i]\Delta\right)^{1-\Delta N_{g,i}} + o(\Delta^L). \tag{S13}$$

VI - Derivation of the Continuous-time PDF of the Disjoint Process: We show a new derivation of the continuous PDF of the disjoint process obtained by Solo. We can obtain this PDF by relating it to the discrete-time joint PDF of Eqs. 9 and S13 as $P\big[\Delta N^*\big] \approx p\big[N^*_{[0,T)}\big]\Delta^L$. Therefore, $p\big[N^*_{(0,T]}\big] = \lim_{\Delta\to 0} \frac{P\big[\Delta N^*\big]}{\Delta^L}$. First, we approximate Eqs. 9 and S13 as follows:

$$P\big[\Delta N^*\big] \approx \exp\left\{\sum_{i=1}^{I}\sum_{m=1}^{M-1} \Delta N^*_{m,i}\left(\log \lambda^*_m[i|H_i]\Delta\right) - \lambda^*_g[i|H_i]\Delta\right\} + o(\Delta^L) \tag{S14}$$

$$= \exp\left\{\sum_{m=1}^{M-1}\sum_{i=1}^{I} \Delta N^*_{m,i}\log \lambda^*_m[i|H_i]\Delta - \lambda^*_m[i|H_i]\Delta\right\} + \ o(\Delta^L), \tag{S15}$$

where we have substituted $\lambda^*_g[i|H_i] = \sum_{m=1}^{M-1}\lambda^*_m[i|H_i]$. Then, we simplify $P\big[\Delta N^*\big]/\Delta^L$ as

$$\frac{P\big[\Delta N^*\big]}{\Delta^L} \approx \frac{\exp\left\{\sum_{m=1}^{M-1}\sum_{i=1}^{I} \Delta N^*_{m,i}\log \lambda^*_m[i|H_i]\Delta - \lambda^*_m[i|H_i]\Delta\right\} + o(\Delta^L)}{\Delta^L} \tag{S16}$$

$$= \frac{\exp\left\{\sum_{m=1}^{M-1}\sum_{i=1}^{I} \Delta N^*_{m,i}\log \lambda^*_m[i|H_i] - \lambda^*_m[i|H_i]\Delta\right\}\Delta^L + o(\Delta^L)}{\Delta^L} \tag{S17}$$

$$= \prod_{m=1}^{M-1} \exp\left\{\sum_{i=1}^{I} \Delta N^*_{m,i}\left(\log \lambda^*_m[i|H_i]\right) - \sum_{i=1}^{I}\lambda^*_m[i|H_i]\Delta\right\} + \frac{o(\Delta^L)}{\Delta^L}. \tag{S18}$$

Finally, we can obtain $p\big[N^*_{[0,T)}\big]$ by passing to the limit:

$$p\big[N^*_{[0,T)}\big] = \lim_{\Delta\to 0}\prod_{m=1}^{M-1} \exp\left\{\sum_{i=1}^{I} \Delta N^*_{m,i}\left(\log \lambda^*_m[i|H_i]\right) - \sum_{i=1}^{I}\lambda^*_m[i|H_i]\Delta\right\} + \frac{o(\Delta^L)}{\Delta^L} \tag{S19}$$

$$= \prod_{m=1}^{M-1} \lim_{\Delta\to 0}\exp\left\{\sum_{i=1}^{I} \Delta N^*_{m,i}\left(\log \lambda^*_m[i|H_i]\right) - \sum_{i=1}^{I}\lambda^*_m[i|H_i]\Delta\right\} \tag{S20}$$

$$= \prod_{m=1}^{M-1} \exp\left\{\int_0^T \log \lambda^*_m(t|H_t)dN^*_m(t) - \int_0^T \lambda^*_m(t|H_t)dt\right\}. \tag{S21}$$

VII - Derivation of the Continuous-time PDF in Terms of the MkPP Representation: We show a new form of the continuous PDF of the disjoint process (Eq. 11) in terms of the MkPP representation. We start with the continuous PDF of Eq. 10 (same as Eq. S21) and re-arrange it to obtain the continuous PDF of Eq. 11, expressed in terms of the MkPP representation.

$$p\big[N^*_{(0,T]}\big] = \prod_{m=1}^{M-1} \exp\left\{\int_0^T \log \lambda^*_m(t|H_t)dN^*_m(t) - \int_0^T \lambda^*_m(t|H_t)dt\right\} \tag{S22}$$

$$= \prod_{m=1}^{M-1} \exp\left\{\sum_{\ell=1}^{L} \log \lambda^*_m(t_\ell|H_{t_\ell})dN^*_m(t_\ell)\right\} \cdot \prod_{m=1}^{M-1} \exp\left\{-\int_0^T \lambda^*_m(t|H_t)dt\right\} \tag{S23}$$

$$= \prod_{m=1}^{M-1} \prod_{\ell=1}^{L} \lambda^*_m(t_\ell|H_{t_\ell})^{dN^*_m(t_\ell)} \cdot \exp\left\{-\int_0^T \sum_{m=1}^{M-1} \lambda^*_m(t|H_t)dt\right\} \tag{S24}$$

$$= \prod_{\ell=1}^{L} \prod_{m=1}^{M-1} \lambda^*_m(t_\ell|H_{t_\ell})^{dN^*_m(t_\ell)} \cdot \exp\left\{-\int_0^T \lambda^*_g(t|H_t)dt\right\} \tag{S25}$$

$$= \prod_{\ell=1}^{L} \left(\frac{\lambda^*_g(t_\ell|H_{t_\ell})}{\lambda^*_g(t_\ell|H_{t_\ell})}\right)^{dN_g(t_\ell)} \prod_{m=1}^{M-1} \lambda^*_m(t_\ell|H_{t_\ell})^{dN^*_m(t_\ell)} \cdot \exp\left\{-\int_0^T \lambda^*_g(t|H_t)dt\right\} \tag{S26}$$

$$= \prod_{\ell=1}^{L} \prod_{m=1}^{M-1} \left(\frac{\lambda^*_m(t_\ell|H_{t_\ell})}{\lambda^*_g(t_\ell|H_{t_\ell})}\right)^{dN^*_m(t_\ell)} \cdot \lambda^*_g(t_\ell|H_{t_\ell})^{dN_g(t_\ell)} \exp\left\{-\int_0^T \lambda^*_g(t|H_t)dt\right\}. \tag{S27}$$

VIII - Strictly-multivariate Version of Algorithm 2: In Algorithm 2, after simultaneity from the fixed rate Poisson process, one uses the ground process to decide whether to reject observations from the Poisson process. If a draw from the Poisson process is not rejected, one subsequently draws from an $M-1$-dimensional multinomial to decide which component of the multivariate point process the accepted event should be assigned to. The above can replicated without having to first draw from the ground process. This is achieved by drawing events from an M-dimensional multinomial. One of the outcomes of this draw would reject the Poisson draw, while the other $M-1$ outcomes would tells to which component of the process the accepted Poisson draw should be assigned.

Algorithm 4 (Thinning): Suppose there exists λ such that $\sum_{m=1}^{M-1} \lambda^*_m(t|H_t) \leq \lambda$ for all $t \in (0, T]$:

1. Simulate observations $0 < t_1 < t_2 \cdots < t_K \leq T$ from a Poisson point process with rate λ.
2. Set $k = 1$.
3. while $k \leq K$
   (a) Draw $m_k \in \{0, \cdots, M-1\}$ from the M-dimensional multinomial distribution with probabilities $\pi_0 = \frac{\lambda - \sum_{m=1}^{M-1} \lambda^*_m(t_k|H_{t_k})}{\lambda}$ and $\pi_m = \frac{\lambda^*_m(t_k|H_{t_k})}{\lambda}$, $m = 1, \cdots, M-1$

(b) if $m_k = 0$, set $dN^*_m(t_k) = 0$ for all $m \in \{1, \cdots, M-1\}$

(c) else, set $dN^*_{m_k}(t_k) = 1$ and $dN^*_m(t_k) = 0$ for all $m \neq m_k$

(d) $dN(t_k)$ is obtained from $dN^*(t_k)$ as in Text S1

(e) $k = k + 1$.

IX - Multivariate Time-Rescaling Theorem and Kolmogorov-Smirnov Plots:

Proposition: *Let $N^*(t) = \{N^*_m(t) : m = 1, \cdots, M-1\}$ be a multivariate point process defined on $[0, \infty)$ with a finite set of components, full internal history H_t, and left-continuous H_t-intensities $\lambda^*_m(t|H_t)$. Suppose that for $m \in \{1, \cdots, M-1\}$ the conditional intensities are strictly positive and that $\Lambda^*_m(t) = \int_0^t \lambda^*_m(\tau|H_\tau)d\tau \to \infty$ as $t \to \infty$. Then under the simultaneous random time transformations:*

$$t \to \Lambda^*_m(t),\ m \in \{1, \cdots, M-1\},$$

the process $\{(N^*_1(t), \cdots, N^*_{M-1}(t)) : t \geq 0\}$ is transformed into a multivariate Poisson process with independent components each having unit rate.

Note: A "multivariate point process" refers to a vector-valued point process with *no* simultaneities. In this terminology, $N^*(t)$ would be considered a "multivariate point process" (by construction) while $N(t)$, as we have defined it, in general would not. According to the proposition, $N^*(t)$ can be transformed into a multivariate point process whose $M-1$ components are independent Poisson processes each having unit rate.

The proposition is a consequence of (a) the fact that the likelihood of $N^*(t)$ is the product of univariate point-process likelihoods, and (b) the time-rescaling result for univariate point processes.

Let $\{\Lambda^*_m(t_\ell)\}_{\ell=1}^{L_m}$ be the sequence obtained by rescaling points of $N^*(t)$ as in the multivariate time-rescaling theorem. There are L_m such points and the L_m's satisfy $\sum_{m=1}^{M-1} L_m = L$, where L is the total number of events from the ground process $N_g(t)$ in the interval $[0, T)$. Now consider the sequence consisting of $\{\tau^m_1 = \Lambda^*_m(t_1)\}$ and $\{\tau^m_\ell = \Lambda^*_m(t_\ell) - \Lambda^*_m(t_{\ell-1})\}_{\ell=2}^{L_m}$, $m \in \{1, \cdots, M-1\}$. According to the multivariate time-rescaling theorem, the τ^m_ℓ's ($\ell \in \{1, \cdots, L_m\}$, $m \in \{1, \cdots, M-1\}$) are mutually independent exponential random variables with mean 1. This is equivalent to saying that the random variables $\{u^m_\ell = 1 - \exp(-\tau^m_\ell)\}_{\ell=1}^{L_m}$, $m \in \{1, \cdots, M-1\}$, are mutually independent uniform random variables on the interval $(0, 1)$. This latter fact forms the basis of a KS test for GOF assessment much like in the case of a uni-variate point process.

Kolmogorov-Smirnov Test: Assume that CIFs $\hat{\lambda}^*_m(t|H_t)$ were obtained by fitting a model to available data. **For each** m, one can use the following KS GOF test to determine whether or not the $\hat{u}^m_\ell$'s are samples from a uniform random variable on the interval $(0, 1)$:

1. Order the $\hat{u}^m_\ell$'s from smallest to largest, to obtain a sequence $\{\hat{u}^m_{(\ell)}\}_{\ell=1}^{L_m}$ of ordered values.

2. Plot the values of the cumulative distribution function of the uniform density defined as $\{b_\ell^m = \frac{l-1/2}{L_m}\}_{\ell=1}^{L_m}$ against the $\hat{u}_{(\ell)}^m$'s.

   If the model is correct then, for each $m \in \{1, \cdots, M-1\}$, the points should lie on the 45-degree line. Confidence bounds can be constructed using the distribution of the KS statistic. For large enough L_m, the 95% and 99% confidence bounds are given by $b_\ell^m \pm \frac{1.36}{\sqrt{L_m}}$ and $b_\ell^m \pm \frac{1.63}{\sqrt{L_m}}$, respectively.

Supplemental Material 2 for "Algorithms for the Analysis of Ensemble Neural Spiking Activity Using Simultaneous-Event Multivariate Point-Process Models" by Ba, Temereanca and Brown

IX - A Multinomial GLM of SEMPPs: We start with the discrete-time PDF of Eq. 9 and and parametrize it so that it becomes a GLM with M-nomial observations and logistic link. We may rewrite the discrete-time PDF $P\big[\Delta N^*\big]$ of Eq. 9 as follows:

$$\prod_{i=1}^{I} \exp\left\{\sum_{m=1}^{M-1} \Delta N^*_{m,i} \log\frac{\lambda^*_m[i|H_i]\Delta}{1-\lambda^*_g[i|H_i]\Delta} + \log(1-\lambda^*_g[i|H_i]\Delta)\right\} \tag{S28}$$

where we have substituted

$$\Delta N_{g,i} = \sum_{m=1}^{M-1} \Delta N^*_{m,i} \tag{S29}$$

and have dropped the terms proportional to Δ^L. From here on, Eq. S28 is treated as a likelihood. That is, ΔN^*_i's are observations rather than random variables.

The following relationships turn the above likelihood into a GLM with M-nomial observations and logistic link:

$$\log\frac{\lambda^*_m[i|H_i]\Delta}{1-\lambda^*_g[i|H_i]\Delta} = \beta'_m x_i, \text{ where} \tag{S30}$$

β_m is a d-dimensional vector of parameters to be estimated from the data, x_i is a vector of covariates/features of the same dimension as β_m and $m = 1, \cdots, M-1$. Obviously, the choice of covariates x_i depends on the problem at hand. In the case of neural data, the covariates are chosen so that they capture the effect of the stimulus as well as history on the observed response(s). The history portion comprises of past observations, while the choice of stimulus covariates choosing depends on the experiment. It is easy to obtain an expression for $\lambda^*_m[i|H_i]\Delta$ as a function of $\beta_1, \cdots, \beta_m$ and x_i:

$$\lambda^*_m[i|H_i]\Delta = \frac{\exp\{\beta'_m x_i\}}{1+\sum_{m=1}^{M-1}\exp\{\beta'_m x_i\}}, \tag{S31}$$

$m = 1, \cdots, M-1$. In the case of a bi-variate point process $N(t)$ ($M = 4$), we may recover the marginal probabilities as

$$\lambda_1[i|H_i]\Delta = \lambda^*_1[i|H_i]\Delta + \lambda^*_3[i|H_i]\Delta, \tag{S32}$$
$$\lambda_2[i|H_i]\Delta = \lambda^*_2[i|H_i]\Delta + \lambda^*_3[i|H_i]\Delta. \tag{S37}$$

X - Approximate GLM: For small Δ, the discrete-time likelihood is the product of $M-1$ uni-variate likelihoods (Eq. S15, Text S1). Assuming $\sum_{m=1}^{M-1}\lambda^*_m[i|H_i]\Delta \propto o(1)$, we may write:

$$\log\lambda^*_m[i|H_i]\Delta \approx \log\frac{\lambda^*_m[i|H_i]\Delta}{1-\sum_{m=1}^{M-1}\lambda^*_m[i|H_i]\Delta}. \tag{S38}$$

If we let log $\lambda^*_m[i|H_i]\Delta = \beta'_m x_i$ in the approximate discrete likelihood (Eq. S15, Text S1) , then the multinomial GLM becomes equivalent to $M-1$ uni-variate GLMs with Bernoulli observations and log link.

For bi-variate point processes ($C = 2$, $M = 4$), the $m = 3$ component is of particular interest as it represents the process of simultaneous occurrences.

Supporting Material 3 for “Algorithms for the Analysis of Ensemble Neural Spiking Activity Using Simultaneous-Event Multivariate Point-Process Models”

by Ba, Temereanca and Brown

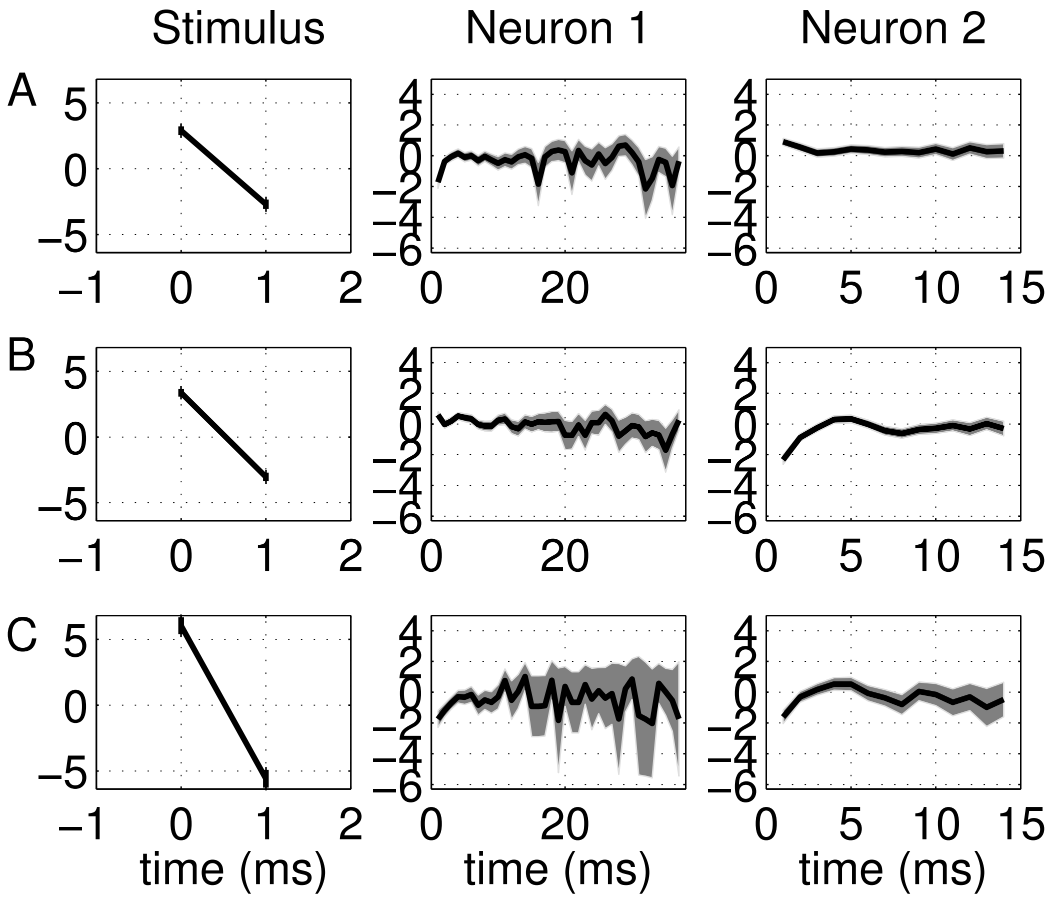

Figure S8. Estimated coefficients of the multinomial GLM model of Eq. 18 for the pair of neurons stimulated with whisker deflection velocity $v =$ 50 mm/s. (A) Coefficients for the '10' event. (B) Coefficients for the '01' event. (C) Coefficients for the '11' event. In all cases, the shaded area surrounding the coefficients correspond to the 95% confidence bounds. Column 1: stimulus coefficients. Column 2 and 3: respectively Neuron 1 and 2 history coefficients. This figure explains why the observed number of simultaneous events (Fig. 1*C*, Column 3) is not as high as the number of non-simultaneous events (Fig. 1*A* and *B*, Column 3), despite the strong stimulus modulation of the simultaneous event observed in Fig. 5. The figure shows that the first few coefficients corresponding to the effect of Neurons 1 and 2 on the probability of simultaneous firing are negative for *both* neurons. This means that if both neurons have just fired, their probability of simultaneous firing within the next 1 to 3 ms decreases significantly. On the other hand, for non-simultaneous events, *only one* of the corresponding set of coefficients is negative. That is, the intrinsic dynamics of Neurons 1 and 2 limit the probability of simultaneous spiking to a greater extent than they limit that of non-simultaneous spiking.

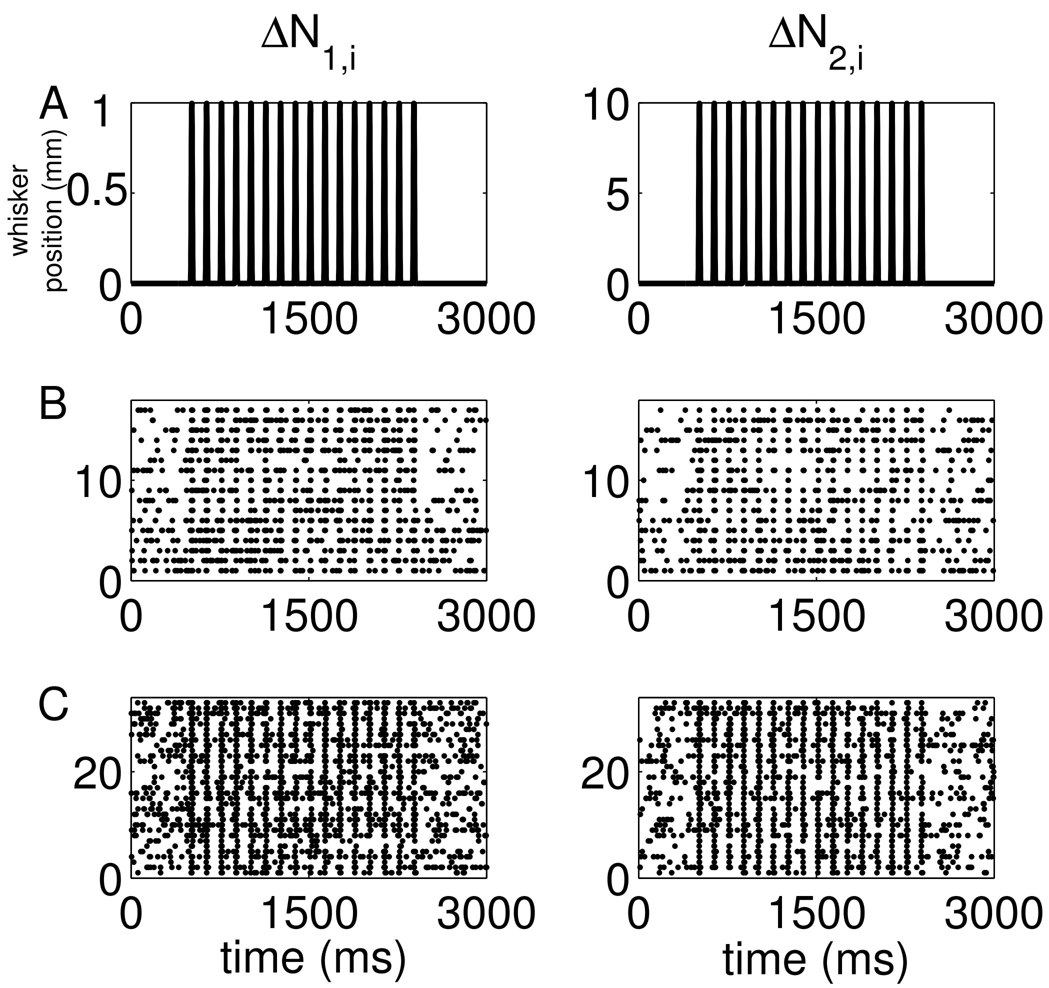

Figure S9. Standard raster plots of the spiking activity of each neuron in a representative pair in response to a periodic whisker deflection of velocity $v =$ 80 mm/s. (A) Stimulus: periodic whisker deflection, (B) 17 trials of training data, (C) 33 trials of test data. The standard raster plots show that the stimulus induces strong modulation of the neural spiking in both the training and test sets. These standard raster plots do not clearly show the effect of the stimulus on joint spiking.

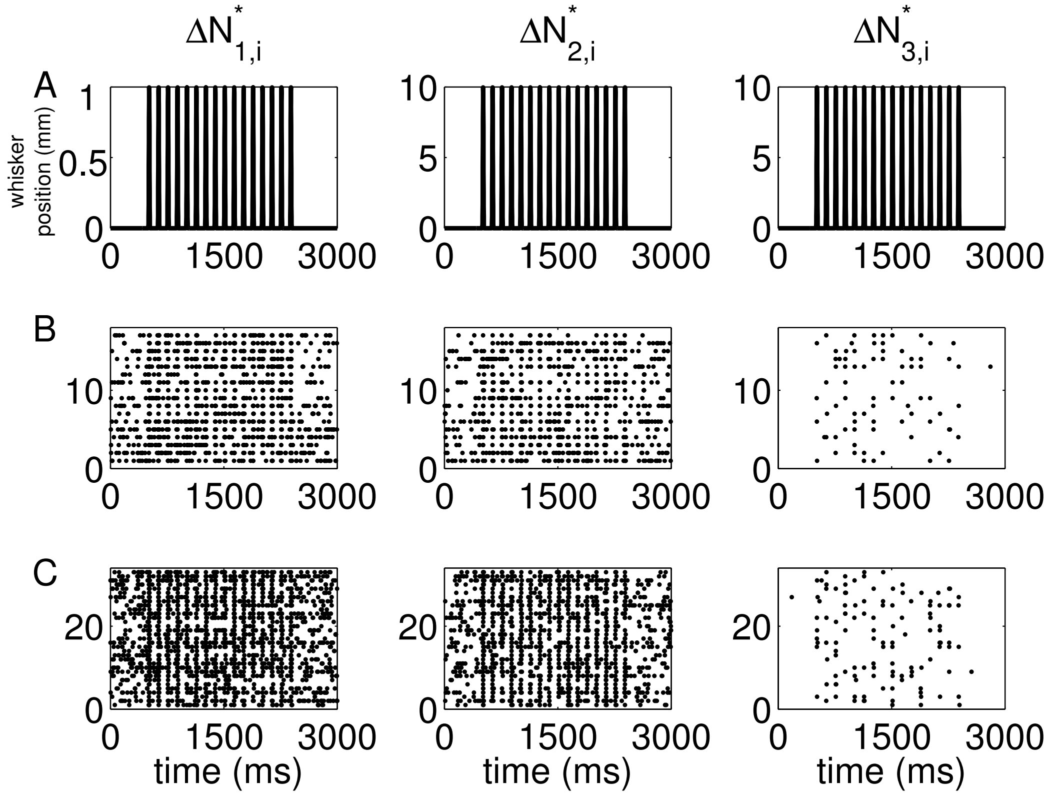

Figure S10. New raster plots of non-simultaneous ('10' and '01', Columns 1 and 2) and simultaneous ('11', Column 3) spiking events for the the neuron pair in Fig. S9. Each column corresponds to one of the components of ΔN^*. (A) Stimulus, (B) 17 trials of training data, (C) 33 trials of test data. The new raster plots of the three components show clearly the effects of the stimulus on non-simultaneous and simultaneous spiking activity. The $\Delta N^*_{3,i}$ component of ΔN^* shows that the joint spiking activity of the two neurons is pronounced.

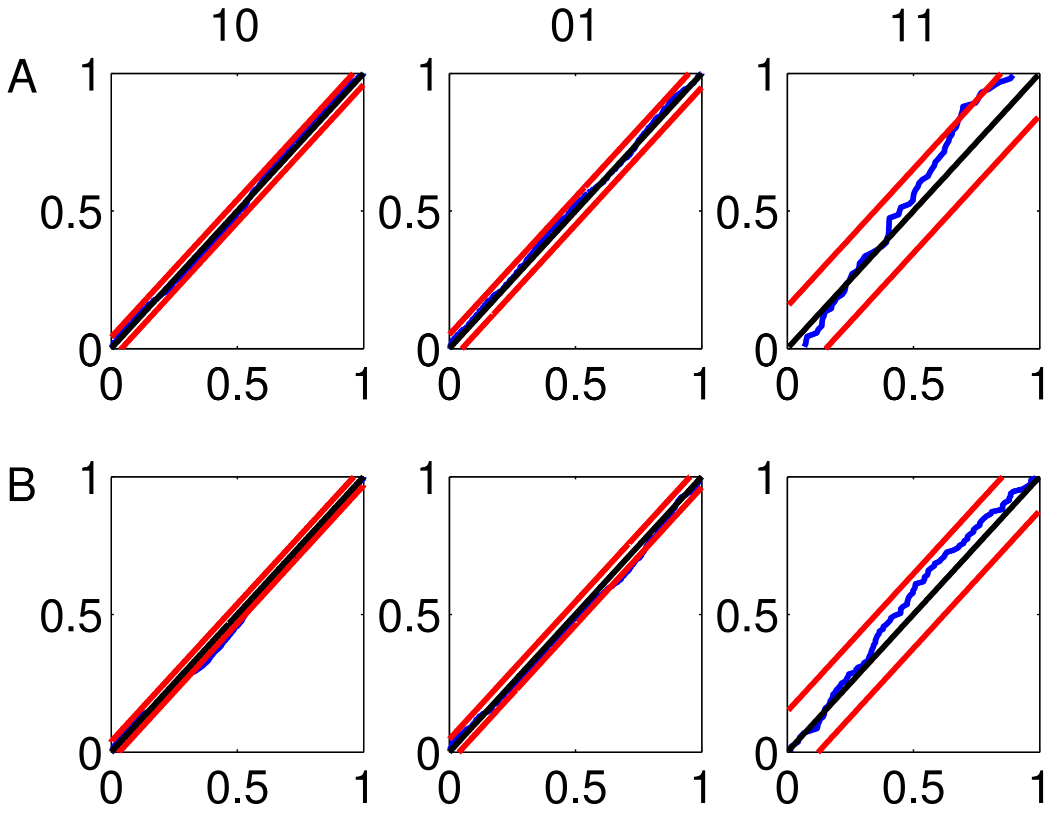

Figure S11. Goodness-of-fit assessment by KS plots based on the time-rescaling theorem. (A) Time-rescaling performance on the training data. (B) Time-rescaling performance on the test data. In both cases, the parallel red lines correspond to the 95% confidence bounds. The KS plots show that the model fits both the training and test data well. The good KS performance on each of the components of ΔN^* demonstrates the model's accurate description of the *joint* process. The performance on the test data demonstrates the strong predictive power of the model.

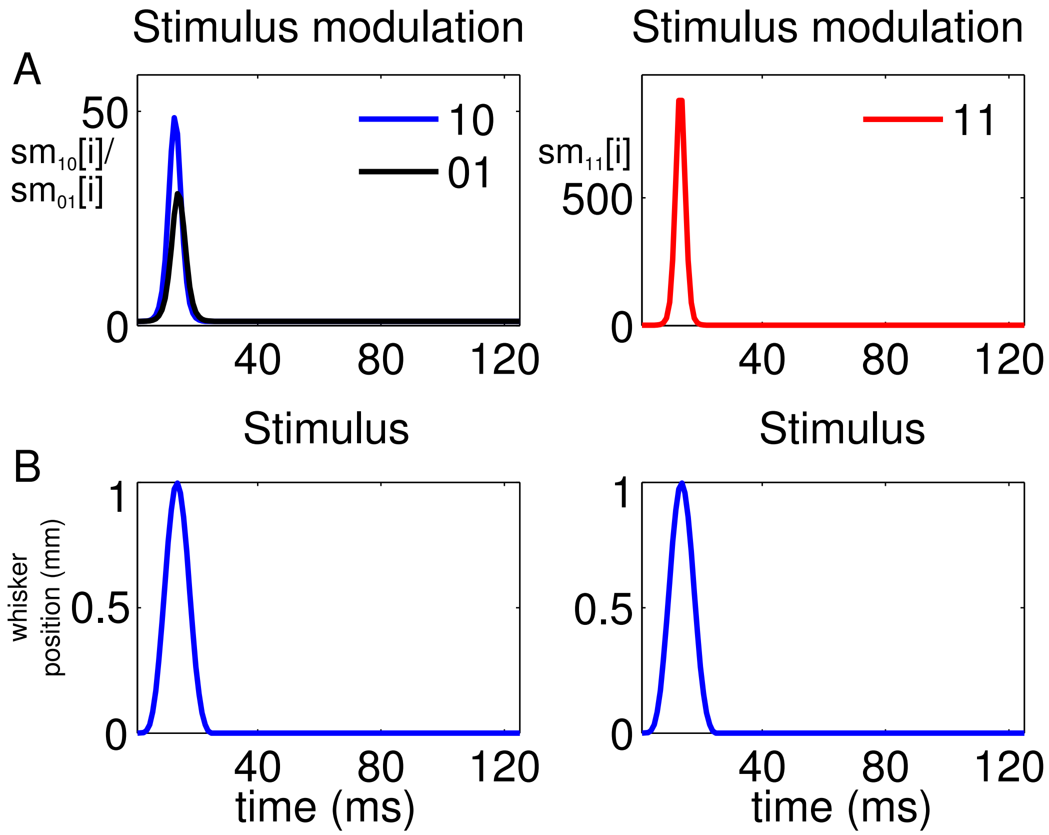

Figure S12. Stimulus modulation of non-simultaneous ('10' and '01') and simultaneous ('11') events, over a single stimulus period. The stimulus modulation of an event is the amount by which the stimulus increases the probability of that event. (A) Left: Stimulus modulation of the non-simultaneous events, ($\exp\{\sum_{j=0}^{J-1} \beta_{m,j}^{(0)} s_{i-j}\}$, $m = 1, 2$). Right: Stimulus modulation of the simultaneous event, ($\exp\{\sum_{j=0}^{J-1} \beta_{3,j}^{(0)} s_{i-j}\}$). (B) Stimulus over a single period. The figure shows that the maximum amount by which the stimulus increases the probability of joint spiking is an order of magnitude greater than that by which the stimulus increases the probability of non-simultaneous spiking. Thus, simultaneous spiking can be attributed for the most part to the administration of the stimulus.

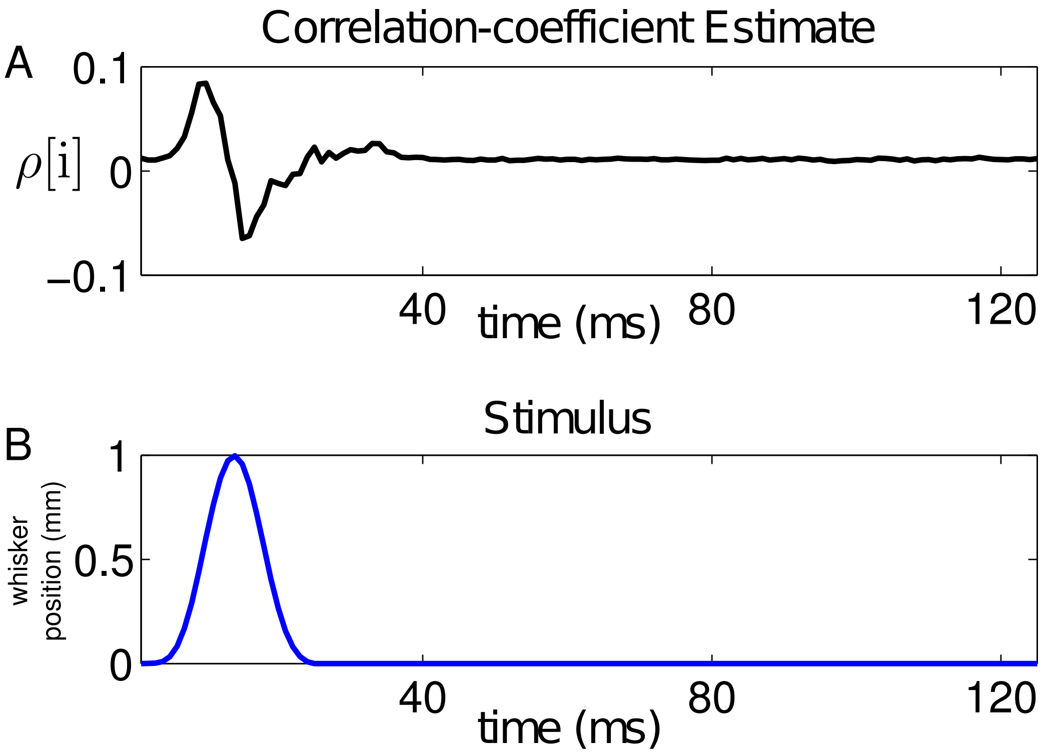

Figure S13. Time-varying assessment of the correlation between the pair of neurons in Fig. S9. (A) Estimate of the correlation coefficient, (B) Stimulus over a single period. The figure demonstrates that the stimulus changes the correlation structure, hence the dependence, between the neurons in the pair at the ms time scale. For this pair, the rising cycle of the stimulus increases the correlation beyond baseline and then decreases it, while the falling cycle makes the neurons negatively correlated, before returning to baseline.

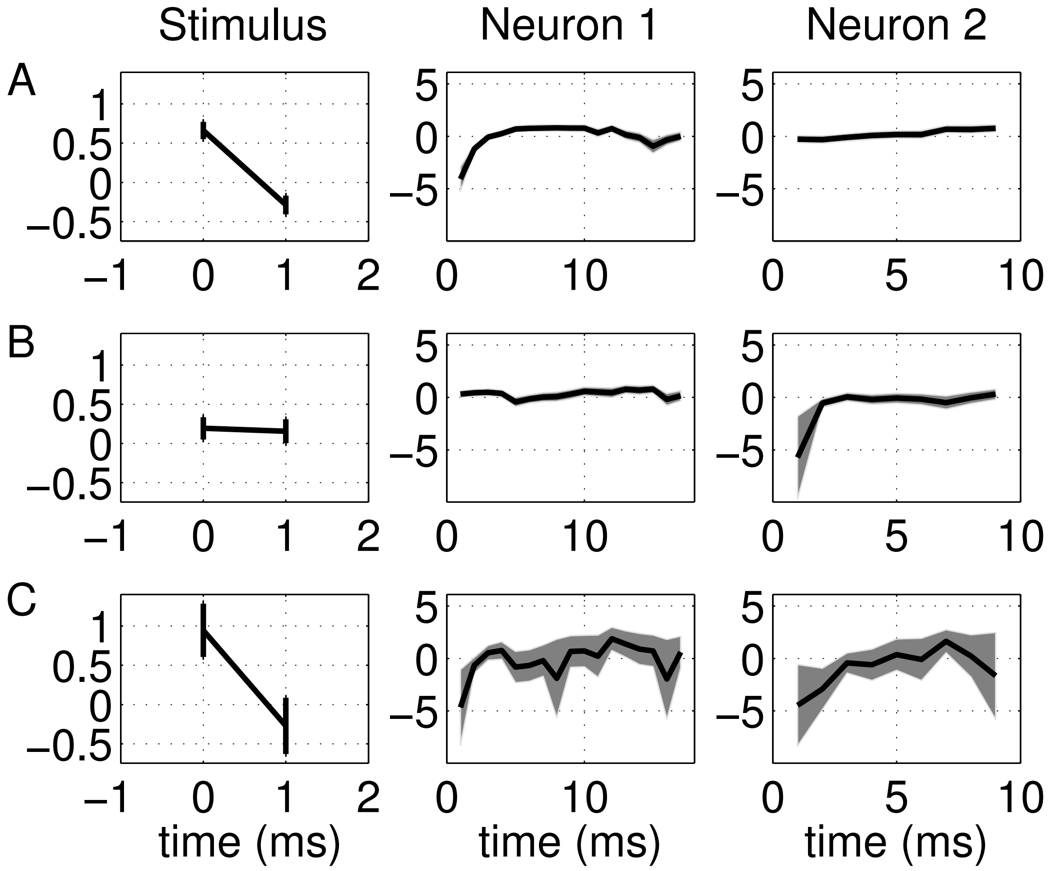

Figure S14. Estimated coefficients of the multinomial GLM model of Eq. 18 for the pair of neurons stimulated with whisker deflection velocity $v =$ 80 mm/s. (A) Coefficients for the '10' event. (B) Coefficients for the '01' event. (C) Coefficients for the '11' event. In all cases, the shaded area surrounding the coefficients correspond to the 95% confidence bounds. Column 1: stimulus coefficients. Column 2 and 3: respectively Neuron 1 and 2 history coefficients. This figure explains why the observed number of simultaneous events (Fig. S10*C*, Column 3) is not as high as the number of non-simultaneous events (Fig. S10*A* and *B*, Column 3), despite the strong stimulus modulation of the simultaneous event observed in Fig. S12. The figure shows that the first few coefficients corresponding to the effect of Neurons 1 and 2 on the probability of simultaneous firing are negative for *both* neurons. This means that if both neurons have just fired, their probability of simultaneous firing within the next 1 to 3 ms decreases significantly. On the other hand, for non-simultaneous events, *only one* of the corresponding set of coefficients is negative. That is, the intrinsic dynamics of Neurons 1 and 2 limit the probability of simultaneous spiking to a greater extent than they limit that of non-simultaneous spiking.

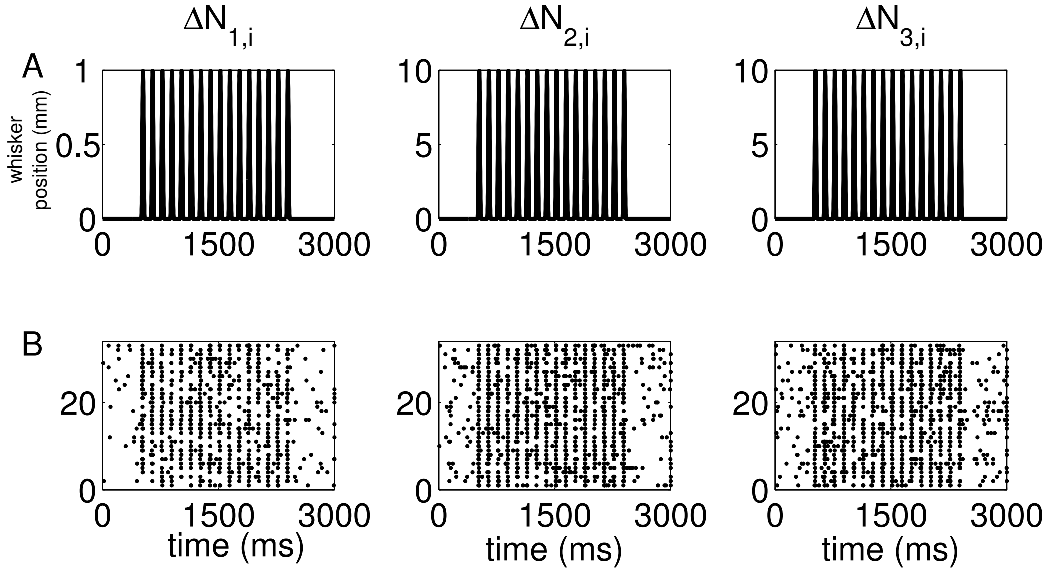

Figure S15. Standard raster plots of the simulated spiking activity of each neuron in a triplet in response to a periodic whisker deflection of velocity v = 50 mm/s. (A) Stimulus: periodic whisker deflection, (B) 33 trials of simulated data. The standard raster plots show that the stimulus induces strong modulation of the neural spiking of each of the three neurons. These standard raster plots do not clearly show the effect of the stimulus on joint spiking. The effect on the stimulus on joint spiking activity is evident in the new raster plots of the disjoint events (Fig. 6).
